# Comprehensive Analysis of microRNAs in Human Adult Erythropoiesis

**DOI:** 10.3390/cells10113018

**Published:** 2021-11-04

**Authors:** Aneesha Nath, Janakiram Rayabaram, Smitha Ijee, Abhirup Bagchi, Anurag Dutta Chaudhury, Debanjan Roy, Karthik Chambayil, Jyoti Singh, Yukio Nakamura, Shaji R. Velayudhan

**Affiliations:** 1 Center for Stem Cell Research (A Unit of InStem, Bengaluru, India), Christian Medical College, Vellore 632002, India; aneesha.micro@gmail.com (A.N.); smitha.ijee@cmcvellore.ac.in (S.I.); abhirup@cmcvellore.ac.in (A.B.); karthik.c@cmcvellore.ac.in (K.C.); 2 Department of Haematology, Christian Medical College, Vellore 632004, India; janakiram.rayabaram@gmail.com (J.R.); anurag.dutta@cmcvellore.ac.in (A.D.C.); debanjan.roy@cmcvellore.ac.in (D.R.); 3 Manipal Academy of Higher Education, Manipal 576119, India; 4 National Centre for Cell Science, University of Pune Campus, Pune 411007, India; jyotis@nccs.res.in; 5 Cell Engineering Division, RIKEN BioResource Research Center, Ibaraki 305-0074, Japan; yukio.nakamura@riken.jp

**Keywords:** microRNAs, small RNA sequencing, erythropoiesis, CD34+ HSPCs, CRISPR-Cas9

## Abstract

MicroRNAs (miRNAs) are small non-coding RNAs, which play an important role in various cellular and developmental processes. The study of miRNAs in erythropoiesis is crucial to uncover the cellular pathways that are modulated during the different stages of erythroid differentiation. Using erythroid cells derived from human CD34+ hematopoietic stem and progenitor cells (HSPCs)and small RNA sequencing, our study unravels the various miRNAs involved in critical cellular pathways in erythroid maturation. We analyzed the occupancy of erythroid transcription factors and chromatin accessibility in the promoter and enhancer regions of the differentially expressed miRNAs to integrate miRNAs in the transcriptional circuitry of erythropoiesis. Analysis of the targets of the differentially expressed miRNAs revealed novel pathways in erythroid differentiation. Finally, we described the application of Clustered regularly interspaced short palindromic repeats-Cas9 (CRISPR-Cas9) based editing of miRNAs to study their function in human erythropoiesis.

## 1. Introduction

Erythropoiesis is a dynamic multi-stage cellular process involving the differentiation of HSPCs to form enucleated red blood cells (RBCs). During erythroid differentiation, cells at each maturation stage are distinct from the other stages in their morphological, cellular, and molecular properties. The earliest lineage-committed progenitors of erythropoiesis are the slowly proliferating burst forming unit-erythroid (BFU-E) cells, which differentiate to rapidly dividing colony-forming unit erythroid (CFU-E) cells that differentiate further into morphologically recognizable proerythroblasts [1,2]. Terminal erythroid differentiation begins with proerythroblasts, which undergo sequential mitoses to form basophilic erythroblasts (baso-E), polychromatophilic erythroblasts (poly-E), and orthochromatophilic erythroblasts (ortho-E), which enucleate to become reticulocytes and eventually into mature RBCs [3,4]. During this process, erythroid cells exhibit a gradual decrease in cell size, hemoglobinization, and chromatin condensation, leading to the final stages of enucleation and expulsion of other cell organelles [5]. Due to the well-orchestrated features of cellular differentiation, erythropoiesis is an important biological process for understanding the general mechanisms of gene regulation in lineage commitment and differentiation [3,4,6,7,8]. An intricate network of transcription factors (TFs), epigenetic modifiers, signaling proteins, growth factors, and long and short non-coding RNAs orchestrate the complex process of erythropoiesis [9,10,11,12].

MicroRNAs (miRNAs), a group of short non-coding RNAs of about 18–24 nucleotides length, regulate gene expression through degradation of mRNAs or translational repression [13,14]. They function as critical modulators of gene expression in various cells, and they are involved in several biological processes, such as cell cycle regulation and cell proliferation, differentiation, and apoptosis [15,16,17,18]. Some miRNAs exhibit tissue-specific or developmental-stage-specific expression and contribute to maintaining tissue identity and function [19]. Several miRNAs have been identified to play significant functional roles in erythropoiesis. These include erythroid lineage determination of the early hematopoietic progenitors, proliferation and terminal differentiation of lineage-committed erythroid progenitors, and enucleation and survival of terminally differentiated erythrocytes [20,21,22,23,24,25,26,27,28,29]. They are also involved in other erythroid cell-associated functions, such as globin gene regulation [30,31,32,33,34], oxidant stress regulation [35,36], and iron absorption [37]. In a few red cell diseases, a correlation between the disease phenotype and expression levels of miRNAs has been reported [38,39,40]. Most of these studies were carried out in mouse and human erythroid cell lines and mouse primary erythroid cells. Very few studies have been carried out using primary human cells to study the roles of miRNAs in human erythropoiesis [21,41].

Deep sequencing of small RNAs provides a highly quantitative estimate of known miRNAs and can discover novel miRNAs within the cells. This method is highly sensitive as it can efficiently detect low abundance miRNAs within the cells [42,43,44]. Small RNA-sequencing in 13 different human tissue samples suggested that there are several novel human-specific and tissue-specific miRNAs (~3500) encoded in the genome [45]. Recently, small RNA sequencing has been applied to study the miRNAs in human erythroid cells [21,41]. These experiments identified miR-4732 as a new member of miR-144/451 cluster critical for erythropoiesis [21] and let7 group of miRNAs and 14q32 cluster miRNAs as stage-specific regulators in adult and fetal erythropoiesis. However, these studies were carried out in mature erythrocytes [21] and in-vitro cultured erythroblasts at an undefined stage of erythroid differentiation [21,41].

Studies in several cell types have revealed that a few cell-specific transcription factors (TFs) are involved in the key functions to maintain cellular identity. In erythroid cells, using chromatin immunoprecipitation-sequencing (ChIP-Seq) analysis to study the genome-wide occupancy of erythroid-specific TFs, a core erythroid transcription network consisting of >300 genes that are positively regulated by GATA-1, SCL, and KLF1 and negatively regulated by PU.1 has been identified [46]. In embryonic stem cells, the cell-specific TFs occupy most of the upregulated miRNAs at the promoters, and they co-occupy with transcription repressors at the silent miRNAs [47] transcription regulatory circuitries integrating TFs, miRNAs, and their targets, which contribute to cell identity and differentiation. Assay for transposase-accessible chromatin using sequencing (ATAC-Seq), a chromatin accessibility test that identifies the genomic regions with active transcription, also helps identify the promoters and enhancers, including those that regulate miRNAs. Although several studies have been carried out on the TF occupancy and miRNA expression in human erythroid cells, the existence of a transcription regulation circuitry involving erythroid TFs and miRNAs has not been established.

A comprehensive analysis of miRNAs in the cells at the distinct stages of progressive erythropoiesis can help understand those miRNAs involved in the transcriptional regulatory mechanisms during erythroid development and differentiation. We carried out ex-vivo erythropoiesis by differentiating CD34+ hematopoietic stem and progenitor cells (HSPCs) to erythroid cells [12,48,49,50], and small RNA sequencing was performed in the cells from distinct stages of erythroid differentiation. Using bioinformatics approaches, we analyzed the targets of the significant miRNAs and identified novel pathways involved in human adult erythropoiesis. Analysis of the occupancy of erythroid TFs at the promoters of the miRNAs could establish transcriptional regulatory circuitries integrating miRNAs and erythroid TFs. Further, the most significantly upregulated miRNAs were functionally validated by gene editing approaches using clustered regularly interspaced short palindromic repeats and CRISPR-associated protein. These miRNAs were knocked out using the CRISPR-cas9 method in HUDEP-2 cells [51], and their effects on cell survival, proliferation, and ability to differentiate were studied.

## 2. Materials and Methods

### 2.1. Ex-Vivo Erythropoiesis

CD34+ HSPCs were isolated from mobilized peripheral blood of a healthy individual using a positive magnetic bead-based selection kit (StemCell Technologies, Vancouver, BC, Canada). In phase I of the culture, 5 × 10^6^ purified CD34+ cells were seeded in a serum-free HSPC expansion medium composed of StemSpan SFEM-II (Stem Cell Technologies, Vancouver, BC, Canada) supplemented with 100 U/mL penicillin,100 µg/mL streptomycin (Thermo Fisher Scientific, Inc., Grand Island, NY, USA), 2 mM L-glutamine (Thermo Fisher Scientific, Inc., Grand Island, NY, USA), 100 ng/mL recombinant human stem cell factor (rh SCF), 100 ng/mL recombinant human fms related tyrosine kinase 3-ligand (rh Flt3-L), 20 ng/mL recombinant human interleukin-6 (rh IL-6), 50 ng/mL recombinant human interleukin-3 (rh IL-3), and 100 ng/mL recombinant human thrombopoietin (rh TPO). After expanding the cells for 5 days in Phase I of the culture, the cells were transferred to Phase II of the culture in Stem Span SFEM-II medium containing 50 ng/mL rh SCF, 40 ng/mL recombinant human insulin growth factor-1 (rh IGF-1), 10 ng/mL IL-3, and 3 U/mL recombinant human erythropoietin (rh Epo). All cytokines were purchased from Peprotech Inc., Rocky Hill, NJ, USA. The cell density was maintained at 0.5 × 10^6^ cells/mL throughout the culture, and the medium was changed every alternate day.

### 2.2. Small RNA Sequencing and Analysis of Differential Expression of miRNAs

The cultured erythroid cells were collected from Day 3 of Phase I and Day 9, Day 11, and Day 14 of Phase II of the erythroid culture. Total RNA was extracted from these cells, and small RNA sequencing was performed using standard protocols. RNA purity was checked using QIAxpert (Qiagen GmbH, Hilden, Germany), and RNA integrity was assessed on TapeStation (Agilent Technologies, Santa Clara, CA, USA) using RNA ScreenTape (Agilent Technologies). After confirming that all the samples had an RNA integrity number of more than 7, the RNA samples were processed for the small RNA library preparation using NEBNext Multiplex Small RNA Library Preparation Kit (New England Biolabs, Ipswich, MA, USA) as per the manufacturer’s protocols. The products were checked for fragment size distribution (140–160 bp) on TapeStation (Agilent Technologies) using High Sensitivity D1000 DNA ScreenTapes (Agilent Technologies) followed by size selection (140–160 bp) on 4% E-Gel EX Agarose Gel (Thermo Fisher Scientific, Rockford, IL, USA). The prepared libraries were quantified using Qubit dsDNA HS Assay Kit (Thermo Fisher Scientific). The obtained libraries were pooled and diluted to a final optimal loading concentration before cluster amplification on the Illumina flow cell (Illumina Inc., San Diego, CA, USA). Once the cluster generation was completed, the cluster flow cell was loaded on Illumina HiSeq 2500 instrument to generate 20 million 50 bp paired-end reads. The bioinformatics analysis to estimate the differential expression of miRNAs was performed with The UEA small RNA Workbench (http://srna-workbench.cmp.uea.ac.uk/, accessed on 2 April 2021) [52] using default settings, allowing one mismatch during the alignments with the small RNA sequence lengths ranging from 16 to 26 nucleotides. The reads were aligned with miRBase version 22.1 (https://www.mirbase.org/ftp.shtml, accessed on 4 April 2021) [53], and normalization of the miRNA reads and differential expression were calculated using DESeq using default parameters.

### 2.3. miRNA Target Analysis

The list of experimentally validated miRNA-target genes was obtained from miRTarBase2020 (http://miRTarBase.cuhk.edu.cn/, accessed on 26 August 2021) [54]. Differential expression of the target genes between HSPCs and erythroid cells was obtained from the published RNA sequencing data in the cells from ex-vivo erythropoiesis (GEO ID: GSE119315). The pathway and gene ontology analysis of the miRNA target genes was performed using Enrichr (https://maayanlab.cloud/Enrichr/, accessed on 22 October 2021).

### 2.4. ChIP-Sequencing and ATAC-Seq Analysis

Previously published ChIP-Seq data on the occupancy of GATA1, EKLF (KLF1), NF-E2, and TAL1 in adult erythroid progenitor cells were obtained from Cistrome Data Browser (http://cistrome.org/db/#/, accessed on 28 August 2021) [55], and the data were viewed in the UCSC genome browser. ATAC-Sequencing data files generated from the erythroid cells from different stages of differentiation (HSPC, CFU-E, pro-normoblasts, and late-basophilic stages) were obtained from a previously published data set (GEO accession: GSE128266) [56] and viewed in the UCSC browser.

### 2.5. gRNA Design

The genomic sequences of the mature miRNAs with additional 20 nucleotides on both sides were obtained from the UCSC genome browser (https://genome-euro.ucsc.edu, accessed on 26 January 2021) to design the gRNAs targeting these sequences using CRISPick (https://portals.broadinstitute.org/gppx/crispick/public, accessed on 26 January 2021). For miRNA editing experiments using single gRNAs, the gRNAs were designed adjacent to Drosha and Dicer miRNA processing sites. For the knockouts with dual sgRNAs (d-sgRNAs), a pair of gRNAs was designed within or flanking the miRNA genomic sequences; wherever possible, one gRNA was designed at the miRNA biogenesis processing sites.

### 2.6. Generation of Single and Dual sgRNA Lentiviral Expression Plasmids

LentiCRISPR v2-miRsg plasmids to express single sgRNAs were generated by cloning gRNAs in LentiCRISPR v2 lentiviral plasmid (a gift from Feng Zhang, Addgene #52961) that contains a Cas9 expression cassette and a BsmBI site to clone the gRNAs [57,58]. pMULE-hU6-miRsg1-hU6-sg2-hCMV-Puro-PGK-eGFP (LVMUsg2PG) lentiviral plasmids to express dual gRNAs were generated using multiple lentiviral expression (MULE) system (a gift from Ian Frew, Addgene #1000000060) [59] (Supplementary Figure S1). The dual gRNAs were cloned separately in D9 and D12 entry plasmids of the MULE system at the BfuAI sites. The final lentiviral vector was generated by LR recombination between the D9 and D12 plasmids with the cloned gRNAs, G8 entry plasmid containing human cytomegalovirus (hCMV) promoter to express puromycin resistance gene, and H8 destination plasmid containing phosphoglycerate kinase (PGK) promoter to express enhanced green fluorescence (eGFP) gene. The entry and the destination plasmids were chosen with compatible attL and attR sites for Gateway cloning. MultiSite LR recombination was performed using the Gateway LR Clonase II Plus Enzyme mix (Invitrogen Corporation, Carlsbad, CA, USA) as per the manufacturer’s protocols.

### 2.7. Culture and Differentiation of HUDEP-2 Cells

HUDEP-2 cells were cultured as previously described [51] with minor modifications in the culture medium, which were composed of StemSpan SFEM-II (Stem Cell Technologies, Vancouver, Canada) supplemented with 1 µM dexamethasone (Sigma-Aldrich, St. Louis, MO, USA), 1 µg/mL doxycycline (Sigma-Aldrich, St. Louis, MO, USA), 50 ng/mL rh SCF, 40 ng/mL rh IGF-1, 3 units/mL rh Epo, 10 ng/mL rh IL-3, and supplemented with 100 U/mL penicillin, 100 µg/mL streptomycin, and 2 mM L-glutamine. Differentiation of HUDEP-2 cells was carried out by a previously described protocol [60]. A total of 2 × 10^5^ cells/mL was seeded in erythroid differentiation medium-1 (EDM-1), containing Iscove’s Modified Dulbecco’s Medium (IMDM) supplemented with glutamax (ThermoFisher Scientific, Grand Island, NY, USA), 100 U/mL penicillin, 100 µg/mL streptomycin supplemented with 2% (*v*/*v*) fetal bovine serum (ThermoFisher Scientific, Grand Island, NY, USA), 3% (*v*/*v*) human AB Serum (MP Biomedicals, Solon, OH, USA), 10 µg/mL insulin (Sigma Aldrich, St. Louis, MO, USA), 3 U/mL heparin (Sigma Aldrich, St. Louis, MO, USA), 200 µg/mL holotransferrin (Sigma Aldrich, St. Louis, MO, USA), 1 ng/mL rh IL-3, 10 ng/mL rh SCF, and 1 µg/mL doxycycline (Sigma-Aldrich, St. Louis, MO, USA). On Day 2, the cells were reseeded at a density of 3.5 × 10^5^ cells/mL in fresh EDM-I. On Day 4, 5 × 10^5^ cells were seeded in EDM-II (EDM-I without doxycycline). On Day 6, the cells were seeded at 1 × 10^6^ cells/mL density in EDM-III (EDM –II with 500 mg/mL of holotransferrin). From Day 8, the cells were maintained at a density of 1 × 10^6^ cells/mL in EDM-IV (EDM-III without rh SCF and rh IL-3) until Day 12 with medium change on alternative days.

### 2.8. Generation of Lentiviruses

Lentiviral vectors were prepared by transiently transfecting HEK 293T cells with the lentiviral expression plasmids (LentiCRISPR v2-miRsg or LVMUsg2PG or pLentiCas9-T2A-BFP), packaging plasmid psPAX2 (Addgene 12260) and envelope plasmid pMD2.G (Addgene 12259) (gifts from Didier Trono) using the Fugene HD transfection reagent (Promega Corporation, Madison, WI, USA), following the manufacturer’s protocols. The viral supernatants were collected and pooled at 48, 60, and 72 h and were concentrated 200 times by ultracentrifugation and frozen as aliquots at −80 °C.

### 2.9. Generation of Cas9-HUDEP-2 Cells

About 0.5 million HUDEP-2 cells were transduced with pLentiCas9-T2A-BFP (Addgene 78547) lentiviruses by spinfection at 2250 rpm for 1.5 hrs in the erythroid culture medium containing 8 µg/mL of polybrene (Sigma-Aldrich, St. Louis, MO, USA). The cells were cultured for 5 days, and the transduced cells were selected with 10 mg/mL blasticidin S HCl (blasticidin) (Life Technologies Corporation, Grand Island, NY, USA) for 3–4 days. Then, the selected cells were expanded in culture for 2 weeks before performing the miRNA knockout experiments.

### 2.10. Lentiviral Transduction for miRNA Editing

About 0.5 million HUDEP-2 or Cas9 HUDEP cells were transduced with LentiCRISPR v2-miRsg or LVMUsg2PG lentiviruses by spinfection at 2250 rpm for 1.5 h in the presence of 8 µg/mL of polybrene (Sigma-Aldrich, St. Louis, MO, USA) and incubated at 37 °C and 5% CO_2_. After 24 h, a complete medium change was done. After 5 days, the HUDEP-2 cells transduced with LentiCRISPR v2-miRsg lentiviruses were selected with 1 µg/mL puromycin (Sigma-Aldrich, St. Louis, MO, USA), and the cells transduced with LVMUsg2PG viruses were flow-sorted for the eGFP^+^ cells. The selected transduced cells were expanded for 8–10 days before terminal differentiation and flow cytometry analysis of CD71 and CD235a expression.

### 2.11. Flow Cytometric Analysis

A sample of 5 × 10^4^ cultured erythroid cells was washed with PBS containing 0.1% BSA (PBSA) and suspended in 100 µL of PBSA containing anti-CD71 APC (dilution 1:50) (BD Pharmingen, San Jose, CA, USA) and anti-CD235a PE-Cy7 (dilution 1:50) (BD Pharmingen, San Jose, CA, USA). The cells were incubated in the dark with the antibodies for 20 min, washed with PBSA, and then analyzed on the Cytoflex LX Flow Cytometer (Beckmann Coulter, Indianapolis, IN, USA). The flow cytometry results were analyzed using FlowJo version 10.0.2 (Treestar, Ashland, OR, USA).

### 2.12. Real-Time PCR Analysis of miRNA Expression

cDNA synthesis of miRNAs was carried out using Mir-X First-Strand Synthesis Kit (Takara Bio Inc., Kusatsu, Shiga, Japan) using 1 µg of total RNA. The forward PCR primers were designed using miRprimer2 (Supplementary Table S1), ref. [61] and mRQ 3′ universal reverse primer supplied with the cDNA synthesis kit. Real-time PCR was carried out using GoTaq qPCR Master Mix (Promega Corporation, Madison, WI, USA).

### 2.13. Globin Chain Analysis by High-Performance Liquid Chromatography (HPLC)

Terminally differentiated erythroid cells were harvested, and globin chain HPLC analysis was performed using a protocol that we previously reported [62].

## 3. Results

### 3.1. Ex-Vivo Erythropoiesis

**Figure 1:**
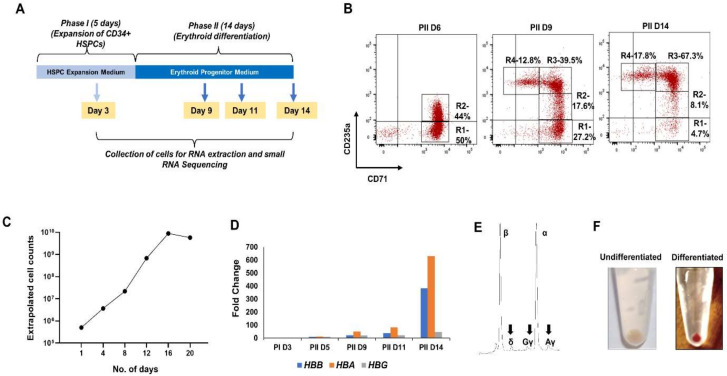
Ex-vivo erythropoiesis to generate cells at different stages of erythroid differentiation. (**A**) Schematic representation of ex-vivo erythropoiesis protocol used in this study. In Phase I of the culture, CD34+ HSPCs were expanded in HSPC expansion medium for 5 days. Subsequently, the cells were cultured in the erythroid progenitor medium for 14 days for progressive erythroid differentiation. Cells were collected from Day 3 and Day 5 of Phase I and Day 9, Day 11, and Day 14 of Phase II of the culture for small RNA expression analysis. (**B**) Flow cytometry analysis of the expression of erythroid surface markers, CD71 and CD235a. (**C**) Extrapolated cell counts showing cell proliferation during ex-vivo erythropoiesis. and (**D**) fold change in the expression of α, β, and γ globin mRNAs on different days of ex-vivo erythropoiesis. (**E**) HPLC analysis of α, β, δ, and γ globin chains in the erythroid cells from the late stage of differentiation. (**F**) The color of pellets of undifferentiated cells from Phase I and terminally differentiated erythroid cells from the end of Phase II of the culture.

Ex-vivo erythropoiesis—differentiation of CD34+ HSPCs to erythroid cells in culture—has been extensively used as a cellular model for studying the transcriptional regulation of human erythropoiesis [12,63,64]. In a two-phase ex-vivo erythropoiesis protocol, Phase I favors the expansion of CD34^+^ HSPCs, and Phase II of the culture allows the robust erythroid differentiation of HSPCs to various erythroid progenitor cells and eventually enucleated reticulocytes [8,65]. Using a modified erythroid differentiation protocol (Figure 1A), we achieved a robust increase in the cell number, up to ~10,000 fold at the end of the culture on Day 20 in Phase II (Figure 1B). Progressive erythropoiesis during Phase II of the culture was assessed by the flow cytometry analysis of CD71^+^CD235a^−^ (R1), CD71^+^CD235a^dim^ (R2), CD71^+^CD235a^+^ (R3), and CD71^dim^CD235a^+^ (R4) populations, which represent CFU-E, pro-, basophilic, polychromatic, and orthochromatic erythroid progenitors, respectively [8,65,66]. On Day 6 in Phase II of the culture, ~50% and 44% of cells were in R1 and R2 populations, respectively. As the cells underwent differentiation, a significant number of cells in R3 and R4 populations were formed. On Day 9 of Phase II of the culture, 39.3% of the cells were in the R3 population, and the remaining consisted of R1 and R2 populations, and on Day 14, 67.3% and 17.8% of the cells were in R3 and R4 populations, respectively (Figure 1C). Quantitative PCR analysis in the cells collected on different days in Phase II of the culture showed a steady increase in the expression of adult globin genes, *HBA* (α-globin) and *HBB* (β-globin) (Figure 1D). Globin chain analysis of the cells from the late stages of ex-vivo erythropoiesis showed high levels of α and β globin chains (Figure 1E). Centrifuged cells from the late stages of ex-vivo erythropoiesis produced red pellets after differentiation (Figure 1F), indicating efficient hemoglobin synthesis in the terminally differentiated erythroid cells. Cell morphology and surface marker expression changes and hemoglobinization in the late-stage erythroid cells suggested that our ex-vivo erythropoiesis protocol is robust for studying the transcriptional regulators of erythropoiesis, including miRNAs at the different stages of human erythropoiesis.

### 3.2. Small RNA Sequencing of Cultured Erythroid Cells

**Figure 2:**
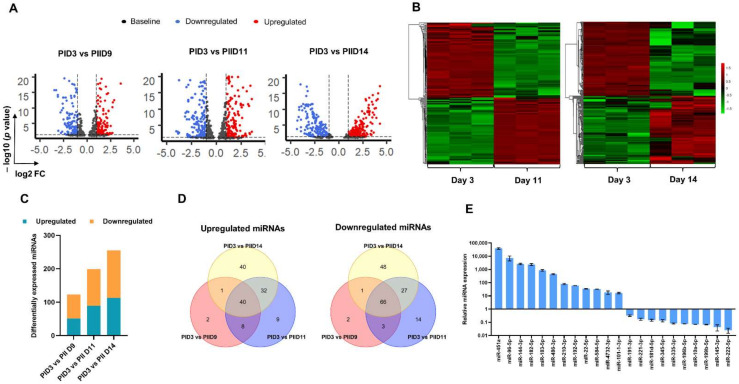
Differential expression of miRNAs at different stages of erythropoiesis. (**A**) Volcano plots showing the differential expression of miRNAs between HPSCs from Day 3 of Phase I of the culture (PID3) and the erythroid cells from Day 9 (PIID9), Day 11 (PIID11), and Day 14 (PIID14) of Phase II of the culture. The log2FC of the miRNAs were plotted against the –log10 of the *p* values. (**B**) Heatmaps of the normalized miRNA reads between PID3 and PIID11 and PID3 and PIID14. The reads from triplicates are shown (R1, R2, and R3). (**C**) The total number of miRNAs with significant differential expression (log2FC > 2 or <−2, *p* < 0.01). (**D**) Upregulated and downregulated miRNAs (log2FC > 2 or <−2, *p* < 0.01) in the cells from different time points of ex-vivo erythropoiesis. (**E**) Validation of the differential expression of miRNAs by quantitative real-time PCR.

To study the expression kinetics of miRNAs in erythropoiesis, we performed small RNA sequencing in the undifferentiated CD34+ HSPCs from day 3 of Phase I of the culture (PID3) and the erythroid cells from the distinct stages of differentiation from Day 9 (PIID9), Day 11 (PIID11), and Day 14 (PIID14) of Phase II of the culture and compared the miRNA profiles in these cells (Figure 1A). In this experiment performed in biological triplicates, there were 846 miRNAs with detectable expression (total reads of miRNA > 100 in all the 12 samples). The principal component analysis of the miRNA reads showed the grouping of the triplicate samples from each time point (Supplementary Figure S2), suggestive of robust reproducibility of the miRNA profiles in the cells collected from a specific stage of differentiation. However, a large number of miRNAs with significant differential expression, both upregulation (up) and downregulation (down), with a gradual increase in their numbers, were observed in the cells from different days of differentiation (Figure 2A). When less stringent criteria to identify the differentially expressed miRNAs were applied (log2-fold change (log2FC) > 1.5 or <−1.5, *p* < 0.05), there were 191 (90 up and 101 down), 307 (152 up and 155 down), and 348 (161 up and 187 down) miRNAs differentially expressed between PID3 HSPCs and PIID9, PIID11 cells, and PIID14 erythroid cells, respectively (Supplementary Figure S3A,B). When the miRNA expression between the undifferentiated HSPCs (PID3 cells) and the differentiated erythroid cells from the two last two time points of differentiation (PIID11 and PIID14 cells) was compared, a total of 409 differentially expressed miRNAs consisting of 193 upregulated (up) and 216 downregulated (down) miRNAs were found (Supplementary Figure S3A,B). Correlating with the previous reports on global downregulation of miRNA expression levels during terminal erythroid differentiation [25,27], there were 89 miRNAs that were downregulated when the cells differentiated from PIID11 to PIID14, but there were a large number of miRNAs with a sharp increase in expression between these two days.

As the cells of a single lineage undergo progressive differentiation in culture, the miRNAs important for the differentiation process would also exhibit robust differential expression. Therefore, we applied more stringent criteria (log2FC > 2 or <−2, *p* < 0.01) to identify the most potential miRNAs involved in the regulation of erythropoiesis (Figure 2B). This analysis yielded 325 differentially expressed miRNAs (149 up and 176 down) between PID3 HSPCs and the PIID11 and PIID14 late-stage erythroid cells (Figure 2C,D and Supplementary Table S2). Out of these, 22 miRNAs exhibited very high differential expression (log2FC > 5 or <−5, *p* < 0.01, total reads > 500), and they consisted of 11 downregulated and 11 upregulated miRNAs (Table 1). These miRNAs included miR-486 and the members of the miR-144-451-4732 cluster, which have been previously reported to be critical for human erythropoiesis [22,67,68]. We identified that the members of the miR-182-183-96 cluster, which have not been reported earlier to have any significant function in human erythropoiesis, are highly upregulated. The levels and the fold change in their expression in the terminally differentiated erythroid cells are found to be similar to those of miR-144 and miR-451.

We performed real-time PCRs to confirm the differential expression of 23 miRNAs with the RNA obtained from the undifferentiated PID3 and PIID14 cells (Figure 2E). Among the differentially expressed miRNAs that we identified, 20 have been reported earlier with various functional roles in mouse or human erythropoiesis (Supplementary Table S3). Altogether, these data showed that the cellular model that we used is suitable for studying the transcriptional regulation involving miRNAs in human erythropoiesis. In addition, a large number of upregulated and downregulated miRNAs, which were not previously reported, were identified in this study. We could identify the previously reported functionally significant miRNAs in erythropoiesis. Furthermore, identifying many differentially expressed miRNAs not previously reported in erythroid differentiation proposes the possibility of previously unknown transcriptional regulatory mechanisms in erythropoiesis.

### 3.3. Erythroid TF Occupancy and Chromatin Accessibility

**Figure 3:**
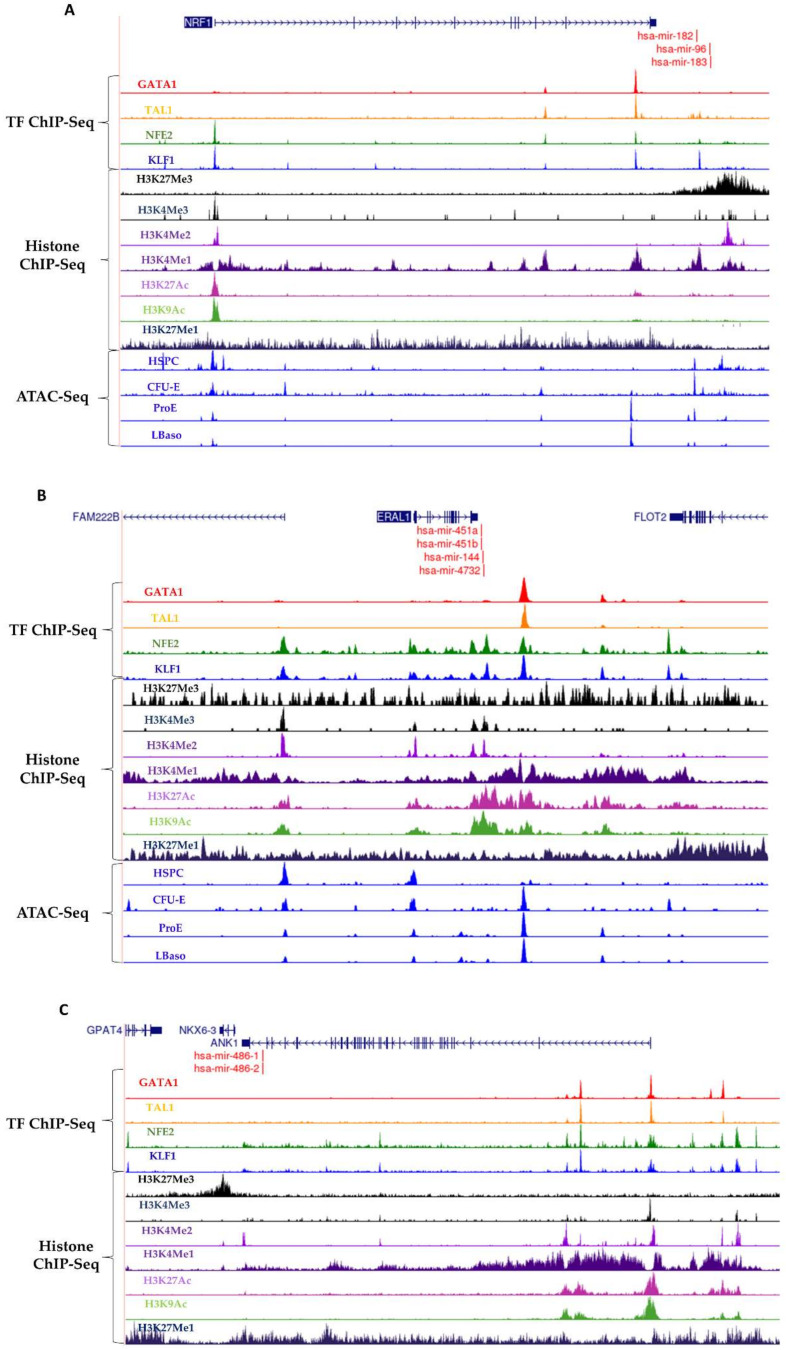
ChIP-Seq density plots of erythroid TFs and histone modifications and ATAC-Seq peaks at the genomic regions of three most upregulated miRNA clusters in human erythropoiesis; (**A**) miR182-183-96 cluster, (**B**) miR 451a-144-4732 cluster, and (**C**) miR486.

Although several studies have been carried out on the TF occupancy and miRNA expression in human erythroid cells, the existence of a transcription regulation circuitry involving erythroid TFs and miRNAs [47] has not been established in these cells. Therefore, we analyzed the occupancy of GATA-1, EKLF (KLF1), NF-E2 and TAL1 TFs at the genomic regions <5 kb upstream of intergenic miRNAs and the transcription start sites of the host genes that harbor the intragenic miRNAs. We selected the most upregulated and downregulated miRNAs for this analysis (Table 1). We did not observe occupancy of the erythroid TFs in the genomic regions near the downregulated miRNA genes. In contrast, all the upregulated miRNAs were found to have strong occupancy of all the four erythroid TFs near their genomic sequences (Figure 3). The selected upregulated miRNAs consisted of miR-182-183-96 (Figure 3A) and miR-144-451-4732 (Figure 3B) clusters and miR-486 (Figure 3C). To confirm transcription activation from the sites occupied by erythroid TFs sites, we also analyzed the ATAC-Seq peaks and found that they co-localize at the promoter regions of the upregulated miRNAs, confirming active transcription from these sites in erythroid cells. The transcription start sites of actively transcribed genes are marked by acetylated H3K27 (H3K27ac) and trimethylated H3K4 (H3K4me3), and active enhancers can be identified by enrichments of both monoacetylated H3K27 (H3K27ac) and monomethylated H3K4 (H3K4me1). We also analyzed these histone modifications and found that these active transcription histone modifications are also present at the genomic regions of the upregulated miRNAs. These data suggested the integration of miRNAs with the erythroid TFs to form transcriptional regulatory circuitries in erythroid cells, as observed in embryonic stem cells [47].

### 3.4. Pathway Analysis

As we obtained a large number of differentially expressed miRNAs, we decided to perform the target gene analysis of these miRNAs and the pathways and molecular functions of these targets. We obtained the list of experimentally validated target genes from miRTarBase 2020 [54] and identified the miRNA and target gene pairs with significant differential expression in the opposite direction. There were 4206 miRNA-gene pairs with significant opposite expression, consisting of 2533 upregulated miRNA-downregulated gene (*Up*miRNA-*Down*gene) and 1673 downregulated miRNA-upregulated gene (*Down*miRNA-*Up*gene) groups. However, there were only 310 miRNAs and 1478 target genes in the list, suggesting multiple miRNAs target the same gene and multiple miRNAs target the same gene in erythroid cells as observed in other cell types.

Out of 563 target genes identified in the *Down*miRNA-*Up*gene group, 14 were targeted by more than 10 miRNAs (range: 10 to 24) (Supplementary Table S4). Of these, 12 genes have been reported to have essential roles in erythropoiesis maintenance and differentiation (Supplementary Table S4). *AGO2*, which is important for erythropoiesis in cellular and mice models, is targeted by 24 miRNAs, suggesting that the expression of *AGO2* is fine-tuned in erythropoiesis by downregulating a large number of miRNAs that target *AGO2*. Others that are involved in erythropoiesis include *CDKN1B* in cell cycle regulation (16 miRNAs), *MKNK2* in HIF-1 signaling and *ERK1*/*ERK2* in MAPK pathways (16 miRNAs), *SLC7A5* in aryl hydrocarbon receptor pathway (16 miRNAs), *TXNIP* in erythropoietin-dependent human erythropoiesis (14 miRNAs), *YOD1* in terminal erythroid maturation (14 miRNAs), *VEGFA* with many functions in hematopoiesis including GATA1 regulation (13 miRNAs), *BCL2L11* in the survival of erythroid cells (12 miRNAs), *TUBB2A* erythroid tubulin regulated by GATA1 and heme (11 miRNAs), *DDIT4* and *CPEB4* in terminal erythropoiesis (10 and 11 miRNAs, respectively), *MYLIP* in inhibiting normal erythropoiesis by proteasomal degradation (10 miRNAs), *SLC1A5* in glutamine transport (10 miRNAs), and *ZBTB7A* in erythroid differentiation factor (10 miRNAs). Out of 914 target genes in the *Up*miRNA-*Down*gene group, 22 genes are targeted by more than 10 miRNAs (range: 10 to 22) (Table S5). Unlike the genes in the *Down*miRNA-*Up*gene pair, only five genes have been reported to have specific functions in erythropoiesis. These include G1/S cell cycle regulators *CCND2* (22 miRNAs) and *CDK6* (19 miRNAs), *RAB32* that affects erythroid cell proliferation and cell death, *HXA10* that inhibits erythroid differentiation (12 miRNAs), and *BCL2* that promotes erythroid cell survival (Table S5).

**Figure 4:**
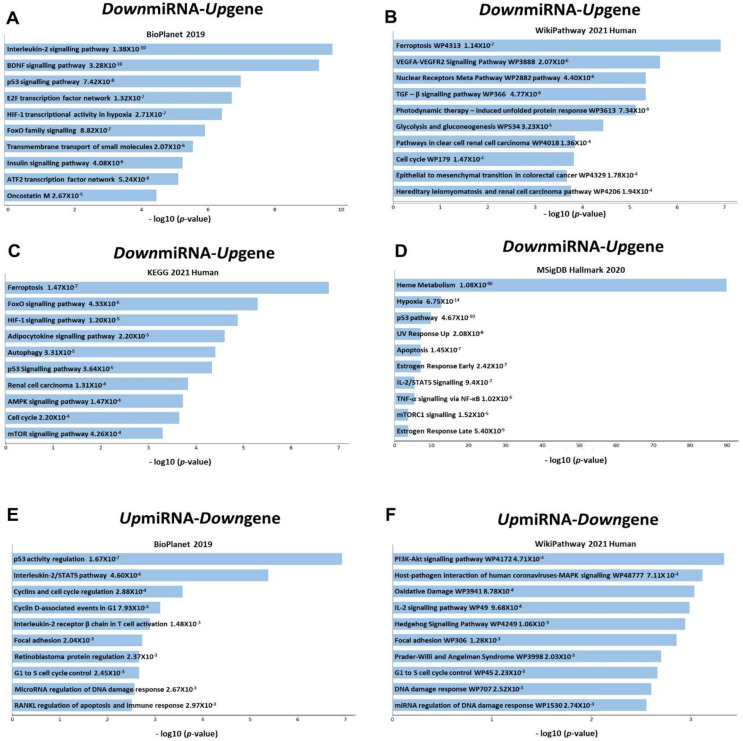
Pathway analysis of the target genes of the differentially expressed miRNAs in erythropoiesis. (**A**–**D**) Significant pathways identified for the genes in the *Down*miRNA-*Up*gene group by (**A**) BioPlanet, (**B**) WikiPathway, (**C**) KEGG, and (**D**) MSigDB Hallmark. (**E**,**F**). Significant pathways associated with the genes in the *Up*miRNA-*Down*gene group by (**E**) BioPlanet and (**F**) WikiPathway.

The pathway analysis of all the genes in the *Down*miRNA-*Up*gene group (Figure 4A–F and Supplementary Figure S4A,B) revealed that the most significant pathway in erythropoiesis regulated by them is ferroptosis, which is a newly identified iron-dependent form of programmed cell death [69] caused by lipid reactive oxygen species (ROS) [70,71,72,73,74]. Although this pathway has been reported in several cell types and biological processes, its role in erythropoiesis has not been described earlier. Other most significant pathways include interleukin-2 signaling, brain-derived neurotrophic factor (BDNF), *p53*, E2-family (E2F) transcription factor network, transforming growth factor-β (TGF-β) signaling, hypoxia-inducing factor-1 (HIF-1) transcriptional activity, FOXO signaling, and vascular endothelial growth factor A (VEGFA)-receptor 2 (VEGFR2) signaling. All these pathways, except BDNF, are involved in different functional aspects of erythropoiesis. The most significant pathways of the *Up*miRNA-*Down*gene group were p53 and interleukin-2/STAT5 pathways, which were also significant in the *Down*miRNA-*Up*gene group.

These results clearly showed that the differentially expressed miRNAs in erythroid differentiation cause modulation of multiple biological pathways crucial for normal erythroid function. Furthermore, despite a few miRNA-target gene pairs, the statistical significance of the pathways identified in this study confirms the role of miRNAs in the erythroid signaling pathways. Such a study has not been performed earlier in human erythropoiesis.

### 3.5. CRISPR-Cas9-Mediated Gene Editing of miRNAs

We selected eight highly upregulated miRNAs, miR-144, miR-451a, miR-4732, miR-182, miR-183, miR-96, and miR-215, and two miRNAs without significant difference in their expression, miR-496 and miR-486, for CRISPR-Cas9-based editing of miRNAs. We employed a recently described protocol that uses gRNAs that target the miRNA biogenesis sites for their efficient knockdown [75]. However, for six miRNAs, we could not successfully design gRNAs with high scores (scores calculated based on the number of off-targets and on-target efficiency) and PAM sites close to the biogenesis processing sites. Due to this technical challenge, we decided to use dual gRNAs that flank the genomic sequences of mature miRNAs (Supplementary Figure S5), hypothesizing that the combination of small insertions and deletions created by individual gRNAs and the deletions created by simultaneous editing with both gRNAs will downregulate the miRNAs more efficiently. Furthermore, to increase the KO efficiency, an effort was taken to include a high-score gRNA with the PAM close to a miRNA biogenesis site in the dual gRNA pair when such a design was possible.

**Figure 5:**
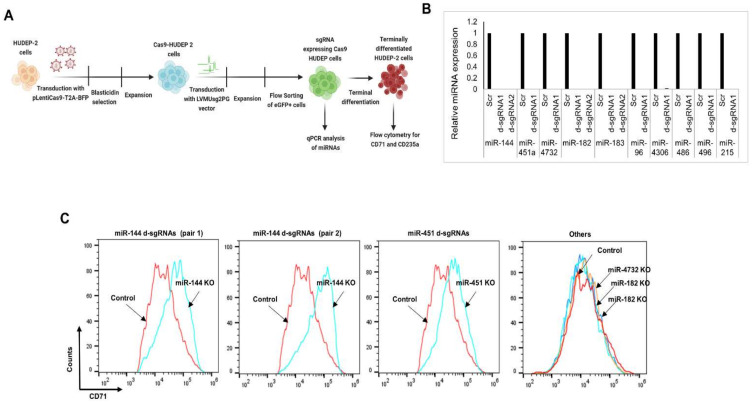
CRISPR-Cas9 editing of miRNAs (**A**) Diagrammatic representation of the CRISPR-Cas9 based editing of miRNAs in HUDEP-2 cells. Cas9-HUDEP2 cells were generated by transduction with pLentiCas9-T2A-BFP lentiviruses, followed by blasticidin selection. The Cas9-HUDEP2 cells were transduced with LVMUsg2PG viruses to express d-sgRNAs that target the miRNAs. The transduced eGFP+ cells were flow-sorted for miRNA knockdown analysis and were terminally differentiated for flow cytometry analysis of CD71 and CD235a expression. (**B**) Real-time PCR analysis of miRNA expression in the Cas9-HUDEP2 cells transduced with LVMUsg2PG lentiviruses that express d-sgRNAs to target the miRNA genes. Scr: scrambled gRNA. d-sgRNA: dual sgRNA. (**C**) Flow cytometry analysis of CD71 expression in the differentiated Cas9-HUDEP-2 after miRNA editing. The results of miRNA-144 KO (with two d-sgRNA pairs) and miR-451 KO (with one d-sgRNA pair) that showed a significant difference in CD71 expression are shown separately from the KO of other miRNAs, miR-4732 (with two pairs of d-sgRNAs), and miR-182 (with one pair of d-sgRNAs). The results for the KO of miR-215, miR-496, miR-486, and miR-4306 are shown in Supplementary Figure S7.

We initially planned to carry out the CRISPR-Cas9 editing of miRNAs by transducing CD34+ HSPCs with lentiviral vectors to express Cas9 and the gRNAs, followed by ex vivo erythroid differentiation. However, as reported earlier [76], we found that the transduction efficiency of CD34+ HSPCs with different Cas9 lentiviral vectors was very low. Therefore, we decided to use HUDEP-2 erythroid progenitor cells [51], which have been extensively used for experiments to study erythropoiesis using several molecular tools, including CRISPR-Cas9-based gene editing [77,78,79]. We generated Cas9-HUDEP-2 cells with a stable expression of Cas9 (Figure 5A), and they were transduced with the LVMUsg2PG lentiviral vectors to express the dual sgRNAs (Supplementary Figure S1). The eGFP^+^ transduced cells expressing gRNAs were differentiated to analyze the effect of miRNA KO in terminal erythroid differentiation. Real-time PCR analysis showed that the expression of the miRNAs targeted by the dual gRNAs was extremely low or undetectable (Figure 5B). As CD71 levels decrease during terminal differentiation, without a notable change in the expression levels of CD235a, we measured the kinetics in the expression of CD71 and CD235a in the dual gRNA-transduced Cas9-HUDEP-2 cells to study the effect of downregulation of the targeted miRNAs in terminal erythroid differentiation.

The results showed that miR-144 KO and miR-451 KO cells had significantly higher levels of CD71 compared to the control cells (Figure 5C) without a change in CD235 expression levels. However, the KO of the other upregulated miRNAs, including miR182, miR-183, and miR-96, with upregulation and expression levels similar to miR-144 and miR-451 in the terminally differentiated cells, did not show any notable difference in the expression levels of CD71 and CD235a (Supplementary Figures S6 and S7). Target analysis using miRTarBase 2020 [54] showed that miR-144 and miR-451 do not target the *TRFC* gene that encodes CD71. Thus, these data clearly showed that although several miRNAs are upregulated during erythropoiesis, only a few of them are involved in erythroid differentiation.

This is the first gene-editing experiment to study the functions of significant miRNAs in human erythropoiesis. The cellular model that we established and the gene-editing strategy using d-sgRNAs for efficient knockouts of miRNAs helps classify the miRNAs into those involved in erythroid differentiation or other functions without tedious experiments using CD34^+^ human primary cells and mice models used in the previous studies. None of the miRNA KO cells showed any significant difference in the cell number throughout the erythroid differentiation for 14 days. Our analysis also indicates that many miRNAs are involved in functions other than erythroid differentiation and survival, requiring further functional characterization experiments.

## 4. Discussion

Several cytokines, signal transduction proteins, TFs, transcription cofactors, epigenetic factors, and non-coding regulatory RNAs, such as miRNAs, are involved at each stage of erythropoiesis. Ex vivo erythropoiesis mimics in vivo erythropoiesis in most of the properties during the stepwise differentiation of HSPCs to terminally differentiated red blood cells. This experimental model has revolutionized the studies on the mechanisms of human erythropoiesis as erythroid culture allows differentiation stage-wise monitoring of erythropoiesis. Several studies have used this model to understand the transcription regulation mechanisms of erythropoiesis using genome-wide occupancy of TFs, gene expression analysis, and gene expression manipulations to characterize the functions of genes that are differentially expressed between the stages of erythropoiesis [12,80,81,82]. However, we realized that a systematic study to understand the profile of miRNA expression at the different stages of adult erythropoiesis has been lacking. Using our ex vivo culture model, we identified a large number of miRNAs that exhibit significant differential expression in adult erythropoiesis. Interestingly, we found a miRNA cluster, miR-182-183-96, with high levels of expression and upregulation, which has not been reported earlier in erythropoiesis.

Analysis of biological pathways regulated by miRNAs in adult erythropoiesis has not been described earlier. We found that several differentially expressed miRNA–target gene pairs are involved in the pathways crucial for erythroid differentiation and functions. One of the most significant pathways of the genes in the *Down*miRNA-*Up*gene group is ferroptosis. Ferroptosis is caused by an accumulation of excessive intracellular iron, depletion of glutathione (GSH), inactivation of glutathione peroxidase 4 (GPX4), and the upregulation of lipid peroxidation in the cells [72,73,83,84]. All these factors that trigger ferroptosis have been reported in erythropoiesis. Although this programmed cell death mechanism has been described in several cell types, the role of ferroptosis in erythropoiesis has not been established earlier.

Further research in understanding the role of ferroptosis in normal and pathological ferroptosis has therapeutical implications. Interestingly, despite the small number of miRNA-target gene pairs, there were 35 genes targeted by more than 10 miRNAs, suggesting the role of miRNAs in fine-tuning the expression of essential proteins involved in crucial functions. Several of these genes are involved in cell cycle regulation, ubiquitination and proteasomal degradation of proteins, and p53 activity regulation. These results clearly showed that the differentially expressed miRNAs in erythroid differentiation cause modulation of multiple biological pathways crucial for normal erythroid function. Despite a few miRNA–target gene pairs, the statistical significance of the pathways identified in this study confirms the role of miRNAs in the erythroid signaling pathways. Such a study has not been performed earlier in human erythropoiesis.

Through extensive ChIP-Seq analysis in embryonic stem cells, it was previously found that the key cell-specific TFs occupy the promoters of most of the preferentially expressed miRNAs, and they form a repressor complex at the promoters of a set of miRNA genes that are silent in embryonic stem cells but expressed in differentiated cells [47]. These data revealed how the key TFs of a cell type promote the miRNA expression program that contributes to cellular identity and differentiation and integrates miRNAs and their targets into an expanded model of the regulatory circuitry controlling cell identity [47]. We tested the existence of such a transcription control mechanism in erythroid cells. We found that the promoters of 15 out of 20 upregulated miRNA genes had occupancy of erythroid TFs. These results suggest that, as previously reported in embryonic stem cells, the erythroid cell-specific TFs integrate the preferentially expressed miRNAs and their targets in the transcription regulatory circuitry in erythroid cells. This finding extends our knowledge of erythropoiesis regulation, and they have therapeutic relevance to red cell diseases.

The widely used methods for miRNA silencing have severe pitfalls [85,86,87]. Recent studies have shown that the function of non-coding RNAs can be disrupted by the CRISPR-Cas9 gene-editing system using the lentiviral expression of sgRNAs [75]. However, in-dels created in the non-coding regions are less likely to produce loss of function phenotypes. Although CRISPR-Cas9 editing of miRNAs using single sgRNAs at the biogenesis processing sites of miRNAs has significantly reduced miRNAs expression, we found that this strategy is challenging due to the lack of high-score gRNAs and unavailability of PAM sites at the biogenesis sites of most of the miRNAs. In a recent study using dual/paired gRNAs, long non-coding RNAs could be deleted with high efficiency [88]. However, the vectors used in this study have not been tested to create deletions of small non-coding RNAs, such as miRNAs. Using the previously described MULE system of cloning different elements of lentiviral vectors [59], we generated lentiviral vectors that express dual sgRNAs (d-sgRNAs) that target miRNAs from two different U6 promoters. Expression of dual gRNAs from this vector in Cas9 expressing HUDEP-2 cells provided high efficiency knock out of miRNA gene expression. Such high-efficiency editing of miRNAs has not been reported earlier, and this method can be used to successfully edit miRNA genes to study their functions in different cell types. We observed that the knockout of miR-144 and miR-451, which were previously reported to be important for mouse erythroid hemostasis [68], causes a reduction in CD71 expression in human erythropoiesis and impaired erythroid differentiation. Further studies using RNA sequencing will help in understanding the mechanisms of ineffective termination observed in the KO cells. As many of the genes in the erythroid cells were regulated by multiple miRNAs, the defect may be compensated by other redundant miRNAs.

In conclusion, we identified several miRNAs with significant differential expression in ex vivo erythropoiesis. Further, we also found the known and new pathways through which these miRNAs function in erythroid cells. In addition, we also described an efficient method for gene editing miRNAs to study their functions. Finally, through the ChIP-Seq and ATAC-Seq analysis, we found that the most upregulated miRNAs in erythroid cells are regulated by erythroid TFs, suggesting the possibility of a transcriptional regulatory circuitry involving TFs and miRNAs in erythroid cells.

## Figures and Tables

**Table 1:** miRNAs with significant differential expression during erythropoiesis (log2FC > 5 or <−5, *p* < 0.01).

miRNA	Total Reads	log2FC			
		PID3 vs. PIID9	PID3 vs. PIID11	PID3 vs. PIID14	
hsa-miR-495-3p	728	−3.07	−5.62	−7.66	Downregulated
hsa-miR-573	557	−0.75	−2.23	−6.94	
hsa-miR-370-3p	8244	−3.67	−5.21	−6.15	
hsa-miR-134-5p	1873	−3.20	−4.57	−5.99	
hsa-miR-543	3293	−4.52	−7.59	−5.36	
hsa-miR-4425	520	−2.63	−3.34	−5.30	
hsa-miR-493-5p	3908	−3.39	−5.22	−5.15	
hsa-miR-654-3p	1477	−3.59	−5.45	−4.58	
hsa-miR-409-3p	6051	−3.06	−5.10	−4.26	
hsa-miR-323a-3p	552	−3.84	−5.63	−3.71	
hsa-miR-1-3p	44,708	−3.83	−5.01	−3.57	
hsa-miR-486-5p	7,025,604	3.44	4.45	5.08	
hsa-miR-183-3p	1725	5.04	6.34	5.26	
hsa-miR-144-5p	275,363	4.64	5.61	5.56	Upregulated
hsa-miR-96-5p	68,467	5.04	5.70	6.10	
hsa-miR-183-5p	435,443	4.54	5.12	6.61	
hsa-miR-182-5p	975,481	4.71	6.04	6.98	
hsa-miR-4732-5p	697	4.92	6.94	7.00	
hsa-miR-375-3p	606	5.29	6.36	7.05	
hsa-miR-144-3p	270,635	5.47	6.96	7.71	
hsa-miR-4732-3p	1288	3.90	6.80	8.81	
hsa-miR-451a	669,963	6.99	9.70	10.21	

(PID3 denotes the cells from Phase I Day 3 of the culture, and PIID9, PIID11 and PIID14 represent the cells from Day 9, Day 11, and Day 14 of Phase II of the culture, respectively).

## Data Availability

The data discussed in this publication have been deposited in NCBI’s Gene Expression Omnibus (GEO) and are accessible through GEO Series accession number GSE185685 (https://www.ncbi.nlm.nih.gov/geo/query/acc.cgi?acc=GSE185685, accessed on 14 October 2021).

## Supplementary Materials

The following are available online at https://www.mdpi.com/article/10.3390/cells10113018/s1, Figure S1. Cloning of d-sgRNAs using LR recombination to generate LVMUsg2PG., Figure S2. Principal component analysis of normalized miRNA reads from the small RNA analysis of the triplicate samples from 4 different time points in ex-vivo erythropoiesis, Figure S3. Differentially expressed with miRNAs in ex-vivo erythropoiesis (log2FC > 1.5 or <−1.5, *p* < 0.05), Figure S4. Pathway analysis, Figure S5. Position of the d-sgRNAs designed near the genomic regions of the miRNAs for CRISPR-Cas9 based editing, Figure S6. CD235a expression in the differentiated HUDEP-Cas9 cells after miRNA downregulation. Figure S7. Flow cytometry analysis of CD71 expression in the differentiated Cas9-HUDEP-2 cells after editing miR-4306, 496, 486, 183, 96, and 215. Table S1. Sequences of the forward primers used for real-time PCR analysis of miRNA expression, Table S2. miRNAs with significant differential expression during erythropoiesis (log2FC > −2 or <2, *p* < 0.01). Table S3: Previously reported miRNAs involved in erythropoiesis identified in this study, Table S4. Molecular functions and pathways of the upregulated genes targeted by multiple downregulated miRNAs, Table S5. Molecular functions and pathways of the downregulated genes targeted by multiple upregulated miRNAs. Table S6. Real-time PCR analysis after miRNA KO in HUDEP2 cells. ΔCt = Ct of target-Ct of U6 (control). ΔΔCt = ΔCt in the KO cells-ΔCt in the control cells. FC = fold change in the expression calculated by the 2^−ΔΔCT^ method. * When the Ct values were undermined due to very low expression, a value of 40 was denoted for calculation. References [9,20,22,32,41,89,90,91,92,93,94,95,96,97,98,99,100,101,102,103,104,105,106,107,108,109,110,111,112,113,114,115,116,117] are referred to in the Supplementary Material file.
