# Supplementary material for: Comprehensive Analysis of microRNAs in Human Adult Erythropoiesis
Source: Cells. 2021 Nov 4;10(11):3018. doi: 10.3390/cells10113018 (PMC8616439; doi:10.3390/cells10113018)
Supplement: Supplementary file 1 [file cells-10-03018-s001.zip › cells-1380865 - Supplementary Material.pdf]

cells MDPI

Supplementary Material

Comprehensive Analysis of microRNAs in Human Adult Erythropoiesis

Aneesha Nath [1], Janakiram Rayabaram [2], Smitha Ijee [1,†], Abhirup Bagchi [1,†], Anurag Dutta Chaudhury [2], Debanjan Roy [2,3], Karthik Chambayil [1], Jyoti Singh [4] Yukio Nakamura [5] and Shaji R. Velayudhan [1,2,*]

[1] Center for Stem Cell Research (A Unit of InStem, Bengaluru, India), Christian Medical College, Vellore 632002, India; aneesha.micro@gmail.com (A.N.); smitha.ijee@cmcvellore.ac.in (S.I.); abhirup@cmcvellore.ac.in (A.B.); karthik.c@cmcvellore.ac.in (K.C.)

[2] Department of Haematology, Christian Medical College, Vellore 632004, India; janakiram.rayabaram@gmail.com (J.R.); anurag.dutta@cmcvellore.ac.in (A.D.C.); debanjan.roy@cmcvellore.ac.in (D.R.)

[3] Manipal Academy of Higher Education, Manipal 576119, India

[4] National Centre for Cell Science, University of Pune Campus, Pune 411007, India; jyotis@nccs.res.in

[5] Cell Engineering Division, RIKEN BioResource Research Center, Ibaraki 6491211, Japan; yukionak@brc.riken.jp

* Correspondence: rvshaji@cmcvellore.ac.in; Tel.: +91-416-2285113

† Equal contribution.

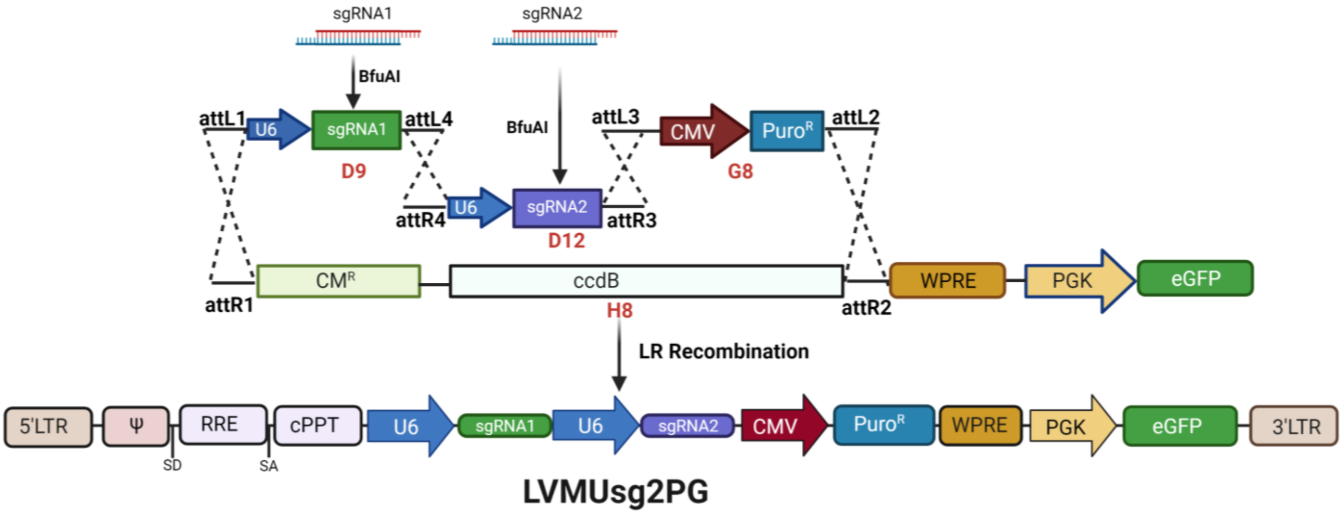

.

Figure S1. Cloning of d-sgRNAs using LR recombination to generate LVMUsg2PG. D9, D12: entry vectors used for cloning sgRNA1 and 2, respectively; G8: entry vector with puromycin resistance gene; H8: destination vector. CMV: human cytomegalovirus (CMV) immediate-early enhancer and promoter; PGK: phosphoglycerate kinase promoter; PuroR: puromycin resistance gene (PuroR), eGFP: enhanced green fluorescence protein, Ψ: psi packaging signal; RRE: rev response element; cPPT: central polypurine tract; LTR: long terminal repeats; WPRE: woodchuck hepatitis virus posttranscriptional regulatory element posttranscriptional regulatory element.

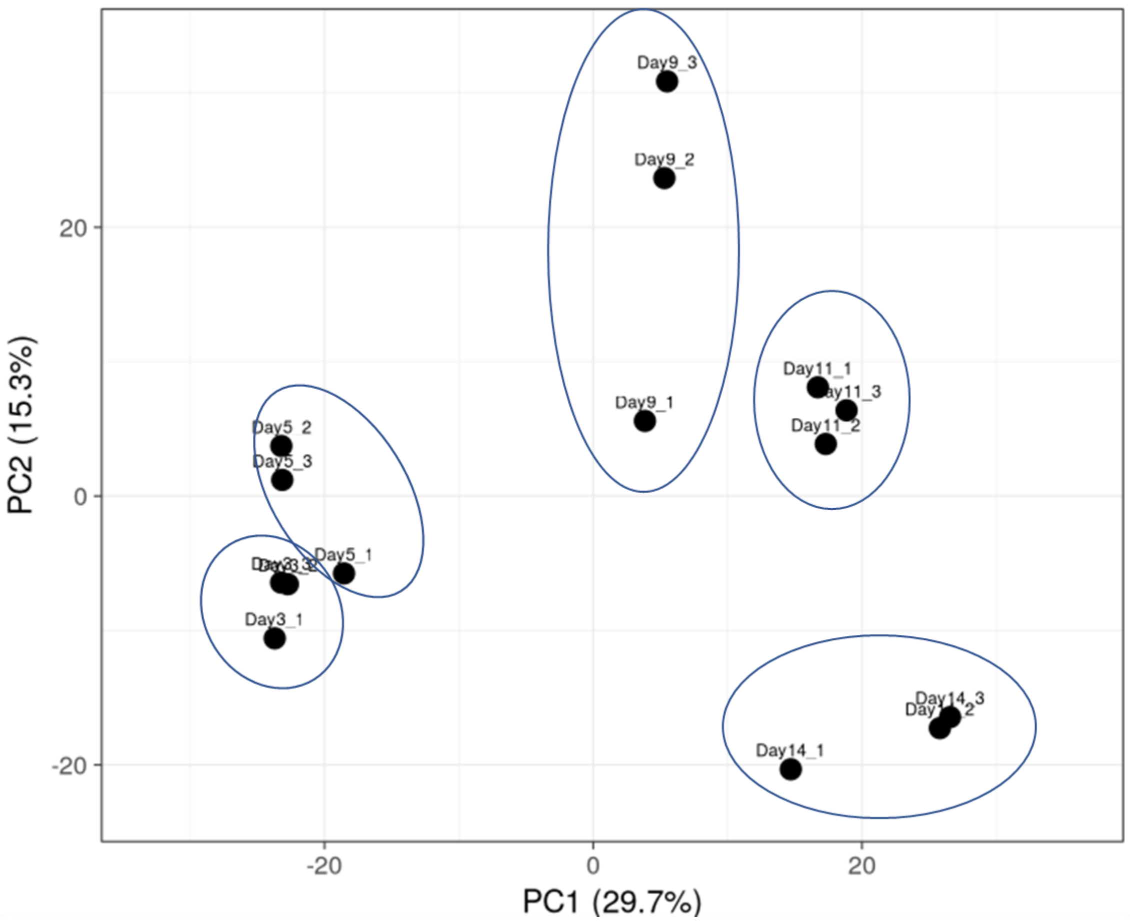

Figure S2. Principal component analysis of normalized miRNA reads from the small RNA analysis of the triplicate samples from four different time points in ex-vivo erythropoiesis. The samples from the same time points are circled.

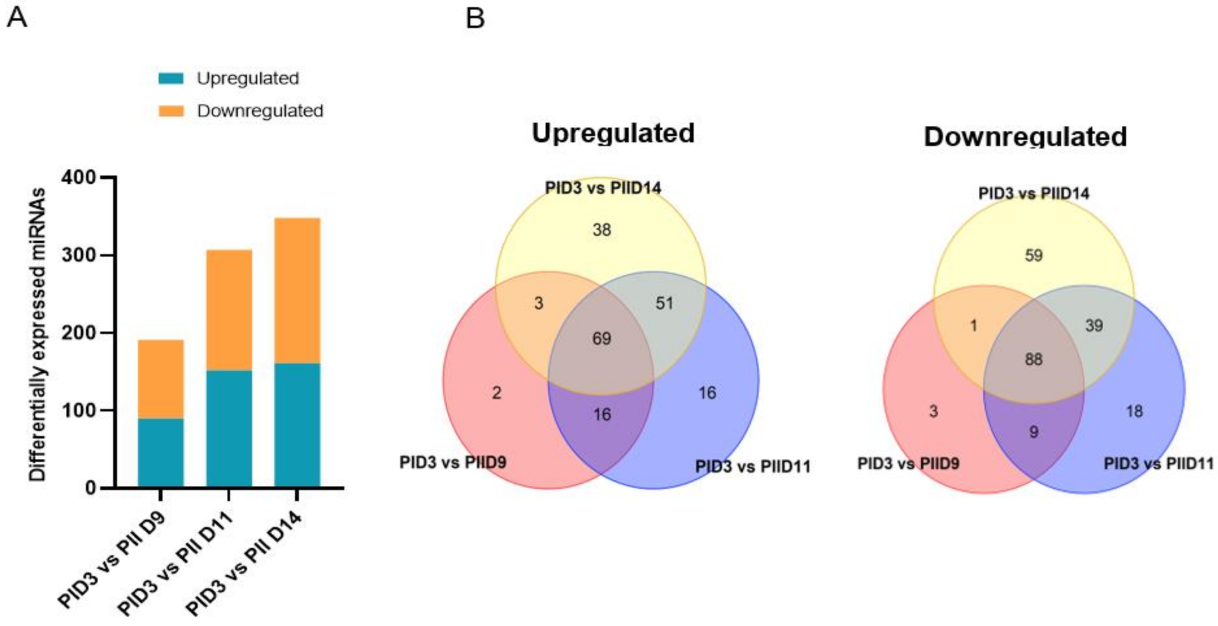

Figure S3. Differentially expressed with miRNAs in ex-vivo erythropoiesis (log2FC>1.5 or <-1.5, p<0.05). (A) The total number of upregulated and downregulated miRNAs (B) Venn diagram showing the number of upregulated and downregulated miRNAs in the cells from different time points of ex-vivo erythropoiesis. PIID9: Phase II Day 9, PIID11: Phase II Day 11, and PIID14: Phase II Day 14.

DownmiRNA-Upgene

A

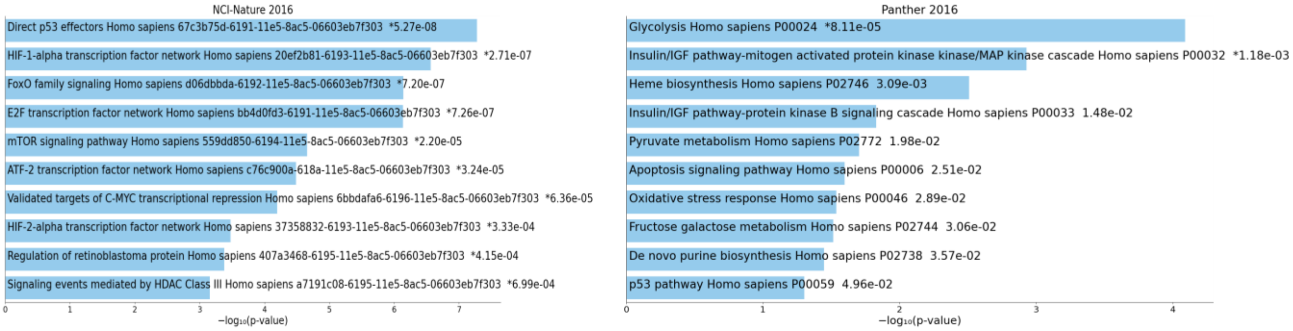

UpmiRNA-Downgene

B

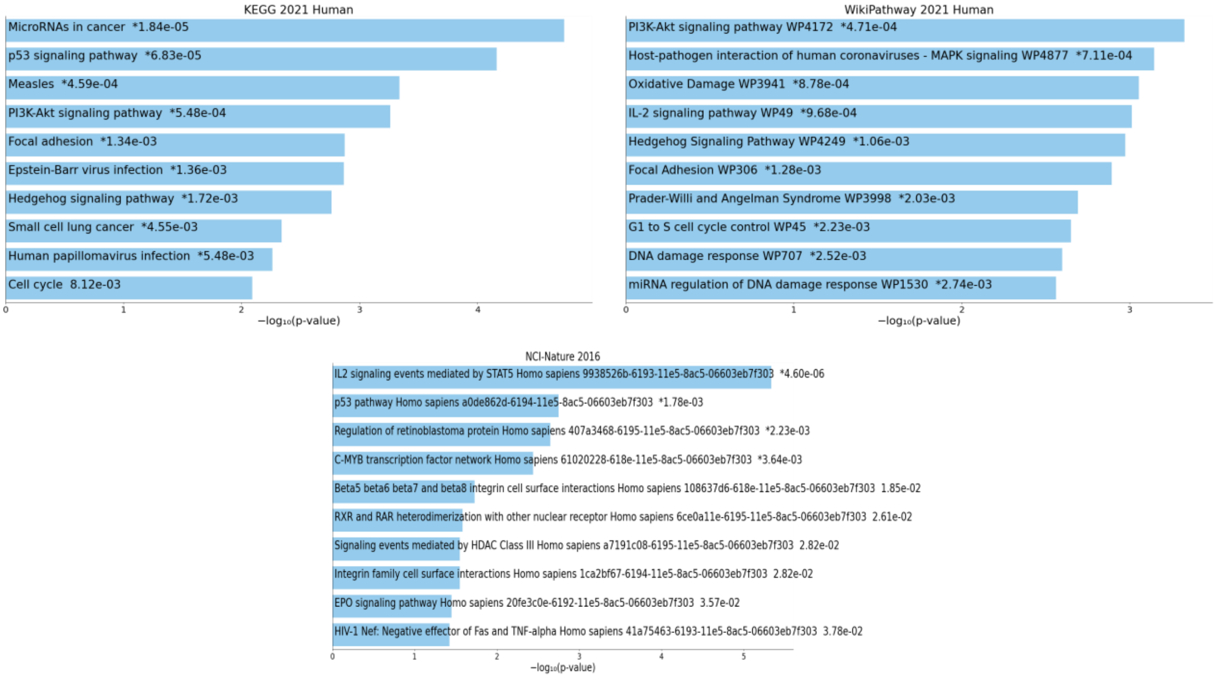

Figure S4. Significant pathways identified for (**A**) the genes in the *Down*miRNA-*Up*gene group using NCI-Nature and Panther databases and (**B**) the genes in the *Up*miRNA-*Down*gene group using KEGG, WikiPathway, and NCI Nature pathway databases.

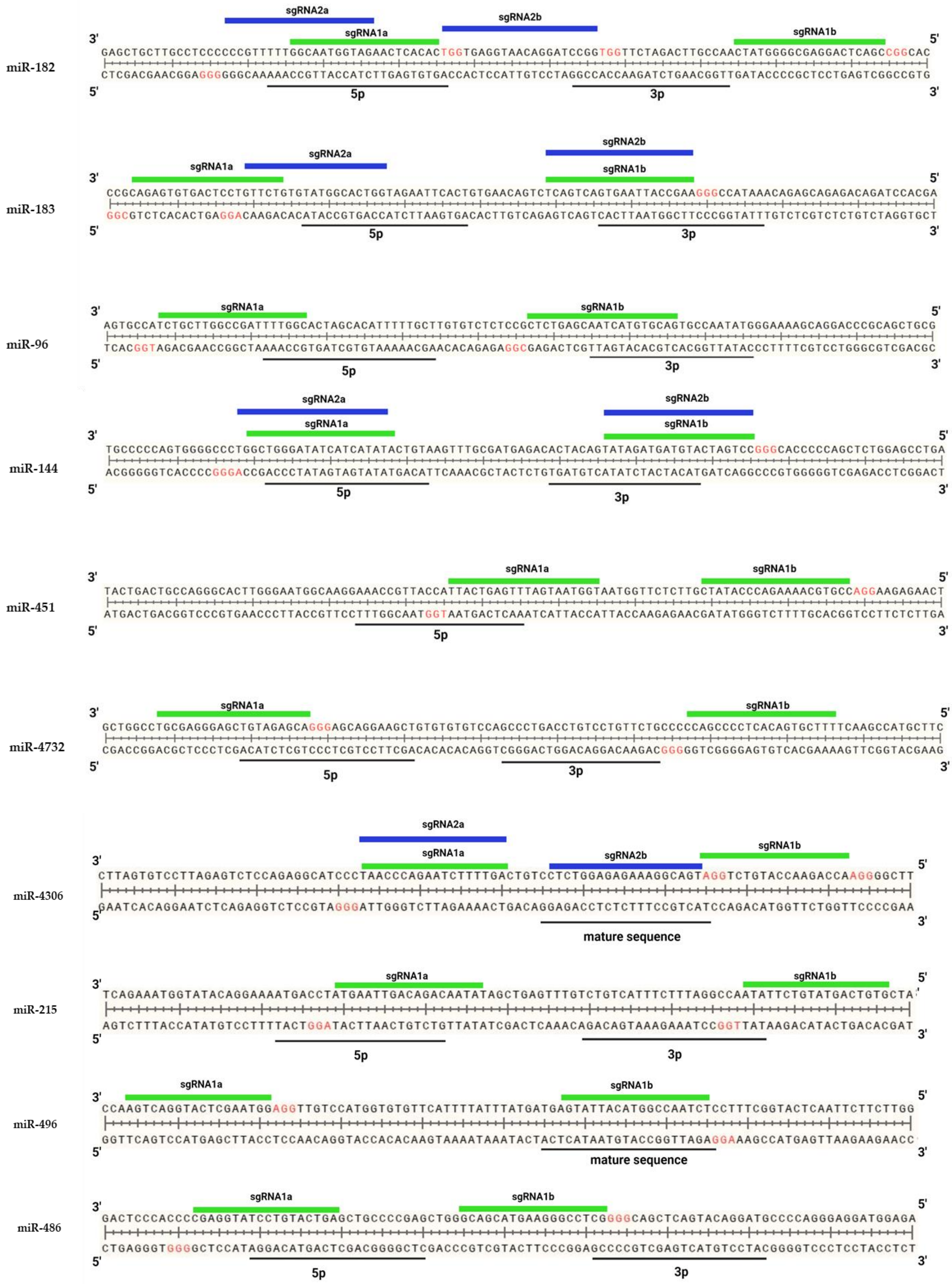

Figure S5. Position of the d-sgRNAs designed near the genomic regions of the miRNAs for CRISPR-Cas9-based editing. sgRNA 1a and sgRNA1b (Pair 1) and sgRNA 2a and sgRNA 2b (Pair 2) denote the pairs of d-sgRNAs designed for targeting the miRNAs. For some miRNAs, Pair 2 could not de designed due to the high off-target scores and low on-target scores and restriction of available PAM sites around the miRNA biogenesis processing sites that are present at the 5′ and 3′ ends of the 5p and 3p mature miRNA sequences.

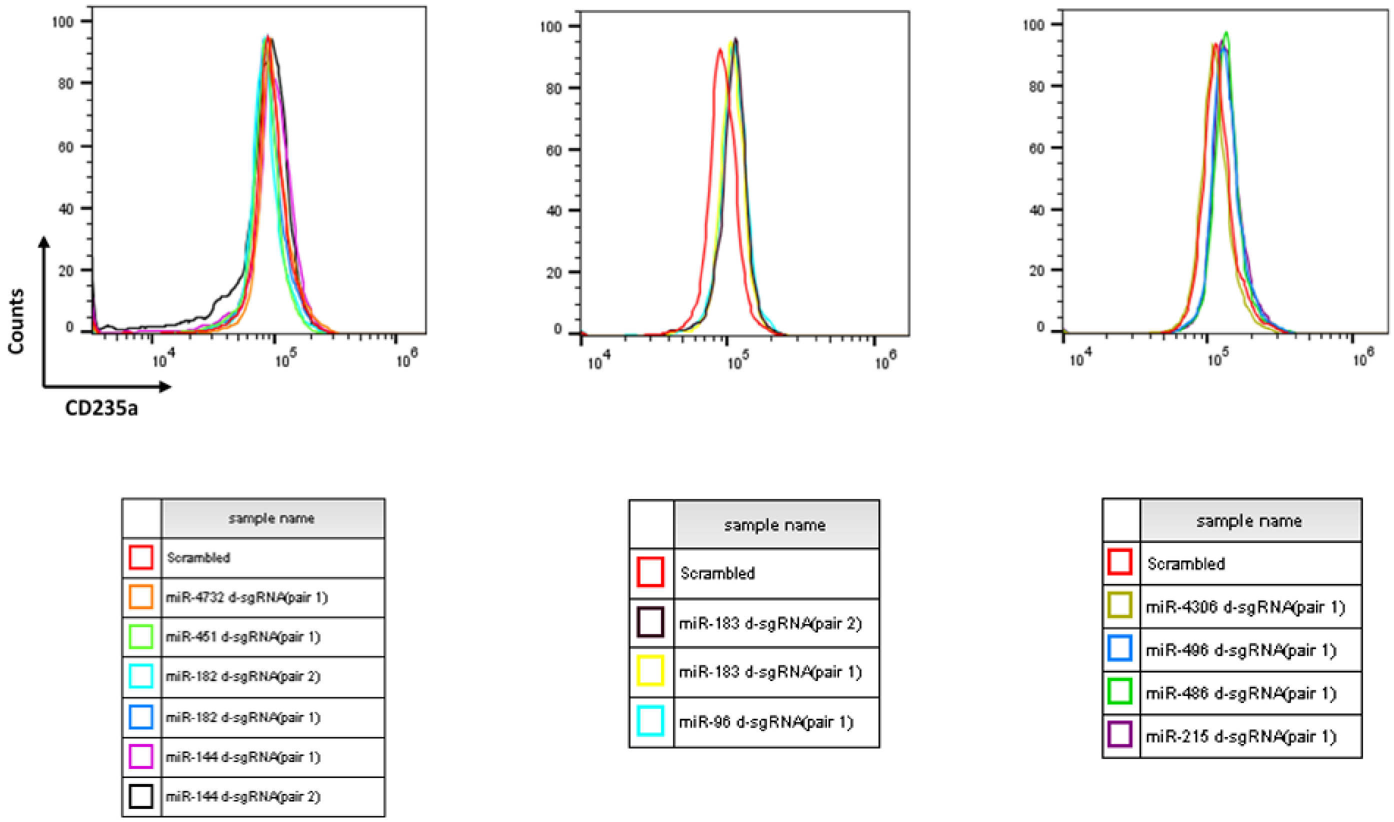

Figure S6. CD235a expression in the differentiated HUDEP-Cas9 cells after miRNA downregulation. The experiments were carried out in three batches. The CD235a expression of the miRNA edited cells was compared to the control cells transduced with scrambled gRNAs.

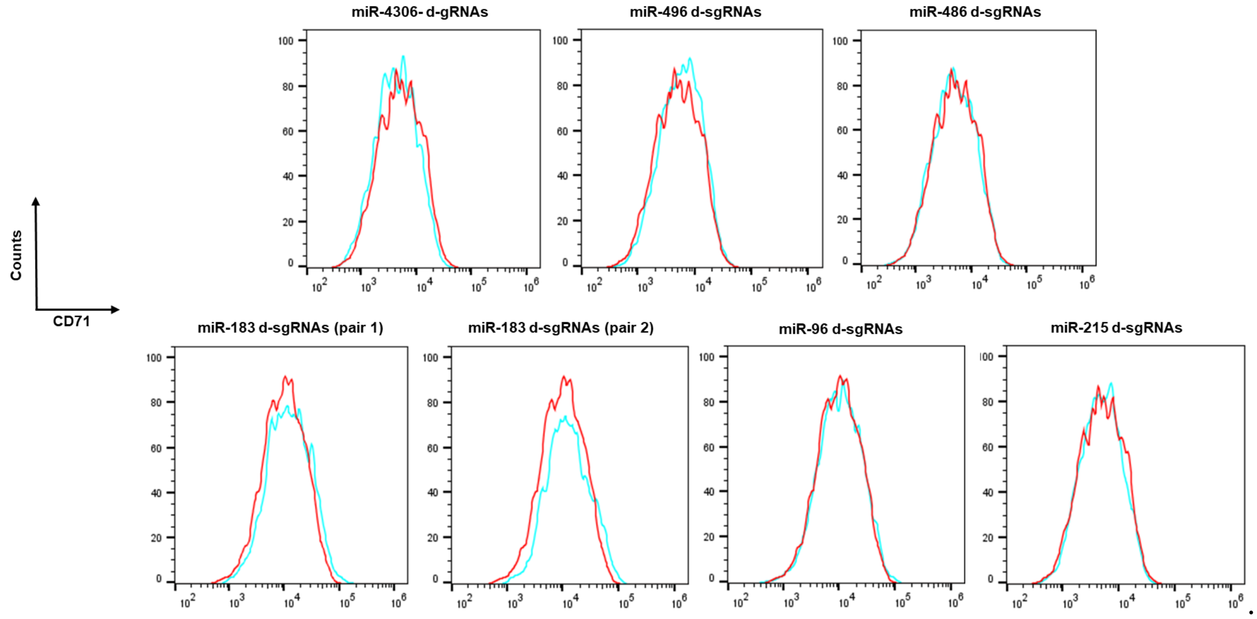

Figure S7. Flow cytometry analysis of CD71 expression in the differentiated Cas9-HUDEP-2 cells after editing miR-4306, 496, 486, 183, 96, and 215. The red lines in the graphs indicate the results from the scrambled d-sgRNA and the blue line indicates the results from the d-sgRNAs targeting the miRNAs. For mir-183, two pairs of d-sgRNAs were used.

Table S1. Sequences of the forward primers used for real-time PCR analysis of miRNA expression.

miRNA	Primer Name	Sequence
hsa-miR-182-5p	miR182-5p-F1	TTGGCAATGGTAGAACTCAC
hsa-miR-182-5p	miR182-5p-F2	GTTTGGCAATGGTAGAACTCA
hsa-miR-183-5p	miR183-5p-F1	GCAGTATGGCACTGGTAGA
hsa-miR-183-5p	miR183-5p-F2	GCAGTATGGCACTGGTAG
hsa-miR-96-5p	miR96-5p-F1	CAGTTTGGCACTAGCACA
hsa-miR-96-5p	miR96-5p-F2	GCAGTTTGGCACTAGCAC
hsa-miR-96-3p	miR96-3p-F2	AATCATGTGCAGTGCCAATATG
hsa-miR-144-5p	miR144-5p-F1	GCGCAGGGATATCATCATATAC
hsa-miR-144-5p	miR144-5p-F2	CGCAGGGATATCATCATATACTGT
hsa-miR-451	miR451-F1	CGCAGAAACCGTTACCA
hsa-miR-4732-5p	miR4732-5p-F1	TGTAGAGCAGGGAGCAG
hsa-miR-4732-5p	miR4732-5p-F2	GTGTAGAGCAGGGAGCA
hsa-miR-4732-3p	miR4732-3p-F1	CCCTGACCTGTCCTGT
hsa-miR-4732-3p	miR4732-3p-F2	GCCCTGACCTGTCCTG
hsa-miR-486-3p	miR486-3p-F1	GGGGCAGCTCAGTACA
hsa-miR-486-3p	miR486-3p-F2	GGGGCAGCTCAGTACAG
hsa-miR-486-5p	miR486-5p-F1	GCAGTCCTGTACTGAGCTG
hsa-miR-486-5p	miR486-5p-F2	CGCAGTCCTGTACTGAG
hsa-miR-496	miR496-F1	CAGTGAGTATTACATGGCCAA
hsa-miR-496	miR496-F2	GCGCAGTGAGTATTACATGG
hsa-miR-215-5p	miR215-5p-F1	CGCAGATGACCTATGAATTGA
hsa-miR-215-5p	miR215-5p-F2	GCAGATGACCTATGAATTGACA
hsa-miR-215-3p	miR215-3p-F1	CGCAGTCTGTCATTTCTTTAGG
hsa-miR-215-3p	miR215-3p-F2	CGCAGTCTGTCATTTCTTTAG
hsa-miR-4306	miR4306-F1	CGCAGTGGAGAGAAAGG
hsa-miR-4306	miR4306-F2	GCAGTGGAGAGAAAGGC

Table S2. miRNAs with significant differential expression during erythropoiesis (log2FC>-2 or <2, p<0.01). (PID3 denotes the cells from the Phase I Day 3 of the culture and PIID9, PIID11, and PIID14 denote the cells from Day 9, Day 11, and Day 14 of the Phase II of the culture, respectively. The fold changes in PIID11 and PIID14 that were considered to decide whether the miRNAs were upregulated or downregulated are underlined).

miRNA	Total reads	Log2FC			Expression
		PID3 vs PIID9	PID3 vs PIID11	PID3 vs PIID14	
hsa-miR-495-3p	728	-3.07	-5.62	-7.66	Downregulation
hsa-miR-573	557	-0.75	-2.23	-6.94	Downregulation
hsa-miR-758-3p	322	-2.63	-7.07	-6.37	Downregulation
hsa-miR-485-3p	228	-3.82	-4.55	-6.36	Downregulation
hsa-miR-370-3p	8244	-3.67	-5.21	-6.15	Downregulation
hsa-miR-134-5p	1873	-3.20	-4.57	-5.99	Downregulation
hsa-miR-551b-5p	386	-5.17	-5.42	-5.58	Downregulation
hsa-miR-4420	113	-1.72	-5.42	-5.49	Downregulation
hsa-miR-433-3p	467	-3.72	-5.82	-5.44	Downregulation
hsa-miR-543	3293	-4.52	-7.59	-5.36	Downregulation
hsa-miR-1910-5p	232	-4.03	-5.98	-5.31	Downregulation
hsa-miR-4425	520	-2.63	-3.34	-5.30	Downregulation
hsa-miR-412-5p	158	-3.53	-5.41	-5.29	Downregulation
hsa-miR-493-5p	3908	-3.39	-5.22	-5.15	Downregulation

hsa-miR-411-3p	273	-4.20	-7.30	-5.15	Downregulation
hsa-miR-1185-1-3p	138	-4.68	-2.77	5.12	Downregulation
hsa-miR-136-3p	184	-2.43	-3.65	-5.10	Downregulation
hsa-miR-493-3p	2876	-2.95	-4.95	-4.98	Downregulation
hsa-miR-379-3p	231	-4.06	-7.06	-4.90	Downregulation
hsa-miR-382-3p	146	-2.81	-6.34	-4.88	Downregulation
hsa-miR-301b-5p	240	-2.80	-5.60	-4.79	Downregulation
hsa-miR-345-3p	209	-0.86	-1.33	-4.75	Downregulation
hsa-miR-889-3p	510	-2.69	-3.13	-4.69	Downregulation
hsa-miR-369-5p	197	-3.02	-4.37	-4.67	Downregulation
hsa-miR-133a-5p	118	-4.18	-4.35	-4.65	Downregulation
hsa-miR-654-3p	1477	-3.59	-5.45	-4.58	Downregulation
hsa-miR-381-3p	6143	-2.51	-3.76	-4.51	Downregulation
hsa-miR-432-5p	739	-2.52	-3.45	-4.49	Downregulation
hsa-miR-127-3p	10875	-2.68	-4.08	-4.49	Downregulation
hsa-miR-485-5p	243	-2.07	-4.26	-4.48	Downregulation
hsa-miR-2115-3p	432	-2.50	-3.64	-4.36	Downregulation
hsa-miR-1914-5p	252	-0.65	-2.55	-4.35	Downregulation
hsa-miR-411-5p	1604	-2.52	-4.15	-4.29	Downregulation
hsa-miR-431-5p	186	-2.77	-3.36	-4.27	Downregulation
hsa-miR-409-3p	6051	-3.06	-5.10	-4.26	Downregulation
hsa-let-7c-3p	610	-1.54	-3.04	-4.22	Downregulation
hsa-miR-760	1085	-0.46	-1.57	-4.13	Downregulation
hsa-miR-222-5p	603	-3.91	-3.76	-4.10	Downregulation
hsa-miR-181b-3p	5124	-2.23	-3.48	-4.09	Downregulation
hsa-miR-654-5p	462	-1.69	-4.18	-4.07	Downregulation
hsa-miR-155-5p	851198	-3.11	-3.75	-4.03	Downregulation
hsa-miR-1250-5p	316	-3.31	-3.98	-4.02	Downregulation
hsa-miR-196a-5p	22557	-2.66	-3.48	-4.00	Downregulation
hsa-miR-455-5p	167	-2.83	-4.61	-3.97	Downregulation
hsa-miR-196b-5p	36664	-2.69	-3.36	-3.94	Downregulation
hsa-miR-3154	215	-0.57	-2.97	-3.88	Downregulation
hsa-miR-382-5p	1636	-2.64	-3.22	-3.87	Downregulation
hsa-miR-551a	230	-2.48	-3.40	-3.83	Downregulation
hsa-miR-1908-3p	893	-0.06	-1.22	-3.79	Downregulation
hsa-miR-124-5p	223	-2.95	-3.33	-3.76	Downregulation
hsa-miR-7111-3p	267	-0.83	-1.50	-3.73	Downregulation
hsa-miR-494-3p	1199	-2.62	-3.17	-3.73	Downregulation
hsa-miR-323a-3p	552	-3.84	-5.63	-3.71	Downregulation
hsa-miR-487b-3p	198	-2.32	-3.27	-3.68	Downregulation
hsa-miR-4741	471	-2.18	-2.66	-3.66	Downregulation
hsa-miR-1-3p	44708	-3.83	-5.01	-3.57	Downregulation

hsa-miR-379-5p	9237	-2.09	-3.48	-3.57	Downregulation
hsa-miR-4802-3p	149	-3.40	-2.53	-3.56	Downregulation
hsa-miR-143-3p	30699	-2.78	-3.81	-3.54	Downregulation
hsa-miR-1257	732	0.57	-0.42	-3.54	Downregulation
hsa-miR-1271-5p	1643	-2.44	-3.11	-3.54	Downregulation
hsa-miR-222-3p	51917	-2.88	-3.04	-3.51	Downregulation
hsa-miR-133a-3p	259	-2.37	-2.57	-3.49	Downregulation
hsa-miR-877-3p	1730	-0.30	-1.41	-3.48	Downregulation
hsa-miR-181a-3p	26090	-2.20	-3.19	-3.46	Downregulation
hsa-miR-329-3p	297	-3.40	-4.72	-3.42	Downregulation
hsa-miR-145-3p	541	-3.43	-3.44	-3.42	Downregulation
hsa-miR-7974	41041	-0.14	-1.45	-3.42	Downregulation
hsa-miR-335-3p	18722	-1.48	-3.03	-3.38	Downregulation
hsa-miR-181c-5p	1189	-2.88	-3.24	-3.38	Downregulation
hsa-miR-99b-3p	26482	-2.33	-3.48	-3.35	Downregulation
hsa-miR-3679-5p	399	-0.85	-1.57	-3.34	Downregulation
hsa-miR-149-5p	1900	-1.58	-2.45	-3.30	Downregulation
hsa-miR-181b-2-3p	1396	-1.46	-2.50	-3.29	Downregulation
hsa-miR-330-3p	19788	-1.35	-1.74	-3.28	Downregulation
hsa-miR-130a-5p	134	-0.11	-1.23	-3.21	Downregulation
hsa-miR-1226-5p	329	0.47	-0.44	-3.20	Downregulation
hsa-miR-145-5p	430	-1.47	-2.61	-3.17	Downregulation
hsa-miR-582-3p	2197	-1.88	-2.95	-3.10	Downregulation
hsa-miR-598-5p	511	-0.69	-1.18	-3.06	Downregulation
hsa-miR-181d-3p	113	-3.43	-3.26	-3.01	Downregulation
hsa-miR-6503-3p	753	-1.10	-2.44	-2.99	Downregulation
hsa-miR-6718-5p	3266	-1.82	-2.42	-2.96	Downregulation
hsa-miR-10523-5p	175	-0.18	-1.94	-2.96	Downregulation
hsa-miR-191-3p	13220	-0.53	-1.54	-2.94	Downregulation
hsa-miR-125b-1-3p	302	-1.44	-3.23	-2.89	Downregulation
hsa-miR-92a-1-5p	61853	-0.49	-0.96	-2.88	Downregulation
hsa-miR-296-3p	8777	0.06	-0.84	-2.85	Downregulation
hsa-miR-181c-3p	3106	-1.89	-2.17	-2.84	Downregulation
hsa-miR-128-1-5p	4476	-0.74	-0.97	-2.84	Downregulation
hsa-miR-338-5p	2296	-2.35	-2.58	-2.82	Downregulation
hsa-miR-1909-3p	119	-1.83	-1.98	-2.79	Downregulation
hsa-miR-6763-5p	125	-0.85	-1.07	-2.76	Downregulation
hsa-miR-4502	210	-0.23	-1.70	-2.75	Downregulation
hsa-miR-146a-5p	856420	-1.88	-2.26	-2.74	Downregulation
hsa-miR-409-5p	216	-3.67	-5.25	-2.74	Downregulation
hsa-miR-26a-1-3p	503	-1.24	-1.59	-2.72	Downregulation
hsa-miR-4645-3p	137	-1.41	-2.76	-2.70	Downregulation

hsa-miR-1273h-3p	7934	-1.60	-2.55	-2.69	Downregulation
hsa-miR-1237-3p	368	0.14	-0.49	-2.68	Downregulation
hsa-miR-5004-3p	147	-1.05	-1.86	-2.65	Downregulation
hsa-miR-6738-3p	219	-1.64	-1.87	-2.65	Downregulation
hsa-miR-1270	259	-3.25	-3.12	-2.62	Downregulation
hsa-miR-345-5p	60850	-0.87	-1.41	-2.57	Downregulation
hsa-miR-221-3p	569114	-2.21	-2.33	-2.53	Downregulation
hsa-miR-1275	31115	0.63	-0.64	-2.53	Downregulation
hsa-miR-130b-5p	52347	-2.35	-3.62	-2.53	Downregulation
hsa-miR-3690	528	-1.77	-3.11	-2.50	Downregulation
hsa-miR-125a-3p	5647	-1.08	-2.12	-2.45	Downregulation
hsa-miR-338-3p	163	-1.79	-3.07	-2.43	Downregulation
hsa-miR-148a-5p	52303	-0.61	-1.70	-2.42	Downregulation
hsa-miR-10a-3p	9117	-1.89	-2.31	-2.41	Downregulation
hsa-miR-766-3p	4958	-0.17	-0.76	-2.39	Downregulation
hsa-miR-378g	15675	-1.20	-1.47	-2.38	Downregulation
hsa-miR-766-5p	844	-1.88	-2.50	-2.37	Downregulation
hsa-miR-1292-5p	1803	-0.78	-1.11	-2.36	Downregulation
hsa-miR-5001-5p	326	-0.26	-0.42	-2.33	Downregulation
hsa-miR-4695-3p	324	-2.15	-1.88	-2.28	Downregulation
hsa-miR-4510	451	-3.12	-2.87	-2.27	Downregulation
hsa-miR-326	3323	-0.63	-1.46	-2.26	Downregulation
hsa-miR-146b-3p	48403	-1.57	-1.98	-2.24	Downregulation
hsa-miR-6789-3p	477	-1.20	-1.64	-2.23	Downregulation
hsa-miR-3940-5p	448	0.14	-0.28	-2.21	Downregulation
hsa-miR-3662	2472	0.21	-1.30	-2.21	Downregulation
hsa-miR-937-3p	815	-1.69	-2.21	-2.21	Downregulation
hsa-miR-503-3p	301	0.94	-0.93	-2.21	Downregulation
hsa-miR-152-5p	348	0.31	-0.12	-2.21	Downregulation
hsa-miR-323b-3p	295	-2.12	-3.35	-2.20	Downregulation
hsa-miR-1249-3p	2980	1.03	0.94	-2.19	Downregulation
hsa-miR-4745-5p	340	-0.21	-1.02	-2.15	Downregulation
hsa-miR-195-3p	310	-0.99	-2.64	-2.13	Downregulation
hsa-miR-629-3p	670	0.01	-0.03	-2.13	Downregulation
hsa-miR-221-5p	8176	-2.09	-2.29	-2.10	Downregulation
hsa-miR-4455	22286	0.39	-1.63	-2.05	Downregulation
hsa-miR-301b-3p	716	-0.91	-1.55	-2.04	Downregulation
hsa-miR-671-5p	16179	0.70	0.41	-2.04	Downregulation
hsa-miR-136-5p	125	-1.45	-2.15	-2.03	Downregulation
hsa-miR-500b-3p	1239	0.47	-0.54	-2.02	Downregulation
hsa-miR-200c-3p	27935	-1.37	-1.62	-2.02	Downregulation
hsa-miR-3620-3p	264	1.55	1.10	-2.01	Downregulation

hsa-miR-1273h-5p	1087	-1.60	-2.74	-2.01	Downregulation
hsa-miR-6798-3p	558	-0.24	-0.64	-2.01	Downregulation
hsa-miR-223-5p	145994	-1.46	-2.59	-1.99	Downregulation
hsa-let-7e-3p	1147	-1.89	-2.89	-1.99	Downregulation
hsa-miR-4707-5p	1125	0.40	-0.46	-2.04	Downregulation
hsa-miR-877-5p	6415	1.42	1.03	-2.03	Downregulation
hsa-miR-181b-5p	278184	-1.52	-1.29	-1.99	Downregulation
hsa-miR-1226-3p	2996	-1.33	-1.50	-2.09	Downregulation
hsa-miR-199b-5p	76015	-1.18	-2.00	-1.90	Downregulation
hsa-miR-301a-5p	6659	-0.78	-1.54	-2.09	Downregulation
hsa-miR-4665-5p	292	-0.98	-1.17	-2.09	Downregulation
hsa-miR-4687-3p	496	0.55	0.17	-2.11	Downregulation
hsa-miR-181d-5p	179291	-1.50	-1.21	-2.10	Downregulation
hsa-miR-200a-5p	251	-0.94	-2.10	-1.82	Downregulation
hsa-miR-11400	499	-0.95	-2.47	-1.82	Downregulation
hsa-miR-200b-5p	134	-2.82	-1.60	-2.02	Downregulation
hsa-miR-29b-1-5p	602	-2.27	-1.63	-2.01	Downregulation
hsa-miR-3911	110	-0.65	-2.67	-1.77	Downregulation
hsa-miR-146b-5p	6174725	-1.41	-2.00	-1.73	Downregulation
hsa-miR-7-5p	1839411	-1.22	-2.00	-1.67	Downregulation
hsa-miR-6787-3p	277	-1.47	-2.27	-1.65	Downregulation
hsa-miR-340-3p	83436	-0.93	-2.01	-1.51	Downregulation
hsa-miR-605-3p	205	-1.64	-2.12	-1.44	Downregulation
hsa-miR-99b-5p	358485	-1.44	-2.32	-1.43	Downregulation
hsa-miR-369-3p	282	-1.67	-3.62	-1.34	Downregulation
hsa-miR-3174	494	-1.26	-2.06	-1.29	Downregulation
hsa-miR-3934-5p	321	-0.86	-2.08	-1.24	Downregulation
hsa-miR-9-5p	1819	-1.72	-2.00	-1.22	Downregulation
hsa-miR-3614-5p	501	-1.28	-2.02	-1.19	Downregulation
hsa-miR-95-3p	129	-2.07	-2.35	-1.13	Downregulation
hsa-miR-100-5p	65413	-1.58	-2.00	-1.12	Downregulation
hsa-miR-10a-5p	810721	-2.10	-2.88	-1.04	Downregulation
hsa-miR-10b-5p	566070	-2.13	-2.95	-0.93	Downregulation
hsa-miR-3681-5p	125	-1.88	-2.17	-0.91	Downregulation
hsa-miR-210-5p	529	1.59	2.31	-0.67	Upregulation
hsa-miR-4443	4504	-1.19	-2.08	-0.67	Downregulation
hsa-miR-98-3p	537	-0.94	-2.03	-0.66	Downregulation
hsa-miR-33b-3p	8370	2.21	2.02	-0.63	Upregulation
hsa-miR-212-5p	511	-1.28	-2.18	-0.54	Downregulation
hsa-miR-3667-5p	599	2.13	2.61	0.41	Upregulation
hsa-miR-4516	209	0.67	2.00	0.58	Upregulation
hsa-miR-664b-5p	162	1.03	2.02	0.63	Upregulation

hsa-miR-4646-3p	267	2.11	2.00	0.86	Upregulation
hsa-miR-193b-3p	445	2.83	2.36	0.99	Upregulation
hsa-miR-193b-5p	275	1.79	2.27	1.18	Upregulation
hsa-miR-185-3p	31052	1.53	2.31	1.26	Upregulation
hsa-miR-6749-3p	322	2.80	3.35	1.37	Upregulation
hsa-miR-532-3p	9012	2.02	2.55	1.41	Upregulation
hsa-miR-362-5p	28304	2.01	2.87	1.53	Upregulation
hsa-miR-501-5p	1069	1.85	3.03	1.55	Upregulation
hsa-miR-636	351	1.68	2.01	1.56	Upregulation
hsa-miR-6777-3p	1561	3.60	3.76	1.57	Upregulation
hsa-miR-124-3p	508	2.13	2.32	1.63	Upregulation
hsa-miR-660-5p	23151	1.09	1.98	1.66	Upregulation
hsa-miR-3675-5p	183	1.51	1.89	1.66	Upregulation
hsa-miR-501-3p	79632	1.28	1.95	1.66	Upregulation
hsa-miR-339-5p	48507	1.16	2.07	1.68	Upregulation
hsa-miR-6833-3p	411	1.51	1.83	1.69	Upregulation
hsa-miR-10395-3p	3625	0.48	1.00	1.70	Upregulation
hsa-miR-6505-5p	722	1.73	2.15	1.73	Upregulation
hsa-miR-627-5p	642	1.36	2.40	1.79	Upregulation
hsa-miR-1294	8246	1.45	2.04	1.82	Upregulation
hsa-miR-218-5p	163	0.85	1.24	2.03	Upregulation
hsa-miR-374a-5p	45420	0.31	0.07	2.02	Upregulation
hsa-miR-12136	23673	2.11	3.77	1.87	Upregulation
hsa-miR-106b-5p	163431	0.71	1.90	2.01	Upregulation
hsa-miR-15b-3p	19377	0.16	0.57	2.00	Upregulation
hsa-miR-301a-3p	421	-0.42	0.56	2.09	Upregulation
hsa-miR-29b-3p	11163	0.58	0.83	2.01	Upregulation
hsa-miR-532-5p	482221	1.38	1.43	2.00	Upregulation
hsa-miR-22-5p	12121	0.79	1.63	2.16	Upregulation
hsa-miR-3942-5p	216	1.52	1.98	2.00	Upregulation
hsa-miR-16-2-3p	226820	0.08	0.54	2.01	Upregulation
hsa-miR-30b-5p	21445	0.64	0.68	2.01	Upregulation
hsa-miR-513a-5p	260	3.22	2.58	2.05	Upregulation
hsa-miR-10400-5p	547	0.49	1.89	2.05	Upregulation
hsa-miR-1278	4375	0.56	0.20	2.05	Upregulation
hsa-miR-5189-5p	471	1.86	1.95	2.06	Upregulation
hsa-miR-324-5p	2974	1.50	2.09	2.07	Upregulation
hsa-miR-548am-5p	109	0.67	1.59	2.07	Upregulation
hsa-miR-4659b-3p	453	2.34	2.16	2.07	Upregulation
hsa-miR-5091	559	1.57	1.23	2.08	Upregulation
hsa-miR-151a-3p	1028070	1.45	1.14	2.08	Upregulation
hsa-miR-4485-3p	695	0.54	1.73	2.09	Upregulation

hsa-miR-1246	987001	-0.21	1.94	2.09	Upregulation
hsa-miR-610	332	1.28	2.04	2.13	Upregulation
hsa-miR-4651	105	2.04	3.78	2.14	Upregulation
hsa-miR-550a-3p	725	2.16	2.65	2.16	Upregulation
hsa-miR-33a-3p	233	0.84	1.90	2.16	Upregulation
hsa-miR-6513-3p	696	0.78	1.26	2.17	Upregulation
hsa-miR-625-5p	5491	0.86	2.01	2.18	Upregulation
hsa-miR-4999-5p	327	0.40	1.42	2.18	Upregulation
hsa-miR-590-5p	133	2.79	3.78	2.19	Upregulation
hsa-miR-500a-5p	804	1.76	3.30	2.20	Upregulation
hsa-miR-548bc	8100	1.87	1.52	2.25	Upregulation
hsa-miR-4488	1494	0.48	1.22	2.27	Upregulation
hsa-miR-3136-5p	172	0.71	1.71	2.28	Upregulation
hsa-miR-548at-5p	295	0.83	2.16	2.29	Upregulation
hsa-miR-454-3p	11839	0.32	2.15	2.29	Upregulation
hsa-miR-185-5p	4525309	2.29	2.68	2.30	Upregulation
hsa-miR-483-5p	127	2.72	5.56	2.30	Upregulation
hsa-miR-4449	361	-0.30	2.87	2.30	Upregulation
hsa-miR-616-5p	221	0.03	0.63	2.30	Upregulation
hsa-miR-6734-5p	518	2.60	3.77	2.30	Upregulation
hsa-miR-550a-3-5p	13173	1.37	1.54	2.31	Upregulation
hsa-miR-502-3p	62502	1.57	2.49	2.35	Upregulation
hsa-miR-5010-5p	412	1.88	2.80	2.36	Upregulation
hsa-miR-1290	219319	-0.13	2.11	2.37	Upregulation
hsa-miR-29c-3p	3840	0.58	1.20	2.39	Upregulation
hsa-miR-151b	642	1.89	2.08	2.40	Upregulation
hsa-miR-3190-3p	327	1.53	1.59	2.41	Upregulation
hsa-miR-3158-3p	13212	1.50	1.71	2.43	Upregulation
hsa-miR-15b-5p	68481	0.63	1.84	2.44	Upregulation
hsa-miR-3912-3p	521	1.51	1.14	2.44	Upregulation
hsa-miR-4301	1568	0.29	2.12	2.48	Upregulation
hsa-miR-6859-5p	269	-0.63	0.33	2.52	Upregulation
hsa-miR-502-5p	268	1.61	3.23	2.52	Upregulation
hsa-miR-4672	202	2.51	3.35	2.52	Upregulation
hsa-miR-548d-5p	9797	0.80	1.99	2.53	Upregulation
hsa-miR-3135a	509	1.31	1.71	2.54	Upregulation
hsa-miR-4511	591	1.84	2.53	2.56	Upregulation
hsa-miR-5690	359	1.25	0.99	2.60	Upregulation
hsa-miR-1255b-5p	2092	1.74	1.97	2.61	Upregulation
hsa-miR-34a-5p	6653	1.89	3.11	2.62	Upregulation
hsa-miR-4791	3836	0.01	1.25	2.67	Upregulation
hsa-miR-339-3p	99074	1.85	2.89	2.76	Upregulation

hsa-miR-5695	231	0.57	1.71	2.76	Upregulation
hsa-miR-660-3p	178	1.65	1.74	2.79	Upregulation
hsa-miR-140-3p	305591	0.77	2.16	2.80	Upregulation
hsa-miR-6505-3p	390	1.90	1.28	2.80	Upregulation
hsa-miR-362-3p	185	0.70	1.67	2.81	Upregulation
hsa-miR-3613-3p	524	2.21	3.06	2.87	Upregulation
hsa-miR-6741-5p	203	1.55	2.29	2.89	Upregulation
hsa-miR-210-3p	9610	2.23	3.35	2.95	Upregulation
hsa-miR-6777-5p	235	4.45	5.27	3.03	Upregulation
hsa-miR-548ay-5p	8349	0.97	2.42	3.03	Upregulation
hsa-miR-935	585	2.97	3.01	3.05	Upregulation
hsa-miR-4497	1538	0.41	1.92	3.05	Upregulation
hsa-miR-151a-5p	17308	2.19	3.05	3.06	Upregulation
hsa-miR-4637	141	1.50	2.57	3.06	Upregulation
hsa-miR-22-3p	242867	0.41	1.61	3.06	Upregulation
hsa-miR-4508	111	0.50	1.68	3.07	Upregulation
hsa-miR-16-5p	316601	0.69	2.15	3.08	Upregulation
hsa-miR-1248	1328	0.46	1.86	3.12	Upregulation
hsa-miR-101-3p	1280346	1.43	1.97	3.13	Upregulation
hsa-miR-3611	349	1.01	1.95	3.13	Upregulation
hsa-miR-182-3p	336	3.40	3.56	3.14	Upregulation
hsa-miR-548c-5p	5641	0.92	2.40	3.15	Upregulation
hsa-miR-584-3p	368	3.45	4.05	3.17	Upregulation
hsa-miR-548b-5p	6127	1.07	2.55	3.18	Upregulation
hsa-miR-4286	3349	2.51	3.58	3.28	Upregulation
hsa-miR-202-5p	234	2.14	2.97	3.29	Upregulation
hsa-miR-486-3p	149729	2.16	1.72	3.44	Upregulation
hsa-miR-4669	226	2.74	2.62	3.46	Upregulation
hsa-miR-3688-3p	1291	0.60	1.77	3.48	Upregulation
hsa-miR-548ab	5103	0.88	2.60	3.49	Upregulation
hsa-miR-624-5p	1183	1.63	2.67	3.52	Upregulation
hsa-miR-548ac	140	0.70	1.47	3.64	Upregulation
hsa-miR-15a-5p	17651	0.71	2.17	3.68	Upregulation
hsa-miR-548an	146	0.31	2.82	3.73	Upregulation
hsa-miR-3163	241	1.40	2.39	3.85	Upregulation
hsa-miR-194-5p	105392	1.84	3.54	3.86	Upregulation
hsa-miR-584-5p	126118	3.74	4.13	3.88	Upregulation
hsa-miR-192-5p	298807	2.05	2.80	3.91	Upregulation
hsa-miR-548au-5p	4141	0.86	2.90	4.02	Upregulation
hsa-miR-548ad-5p	5282	0.92	3.01	4.08	Upregulation
hsa-miR-215-5p	28646	2.05	2.78	4.26	Upregulation
hsa-miR-3200-3p	423	3.44	4.49	4.34	Upregulation

hsa-miR-4448	9924	2.22	3.29	4.37	Upregulation
hsa-miR-3135b	17279	2.35	2.37	4.42	Upregulation
hsa-miR-7976	2166	2.30	3.71	4.91	Upregulation
hsa-miR-4503	137	2.32	4.65	5.08	Upregulation
hsa-miR-486-5p	7025604	3.44	4.45	5.08	Upregulation
hsa-miR-183-3p	1725	5.04	6.34	5.26	Upregulation
hsa-miR-144-5p	275363	4.64	5.61	5.56	Upregulation
hsa-miR-96-5p	68467	5.04	5.70	6.10	Upregulation
hsa-miR-190a-5p	292	3.63	4.83	6.34	Upregulation
hsa-miR-183-5p	435443	4.54	5.12	6.61	Upregulation
hsa-miR-182-5p	975481	4.71	6.04	6.98	Upregulation
hsa-miR-4732-5p	697	4.92	6.94	7.00	Upregulation
hsa-miR-375-3p	606	5.29	6.36	7.05	Upregulation
hsa-miR-7155-3p	175	1.36	5.20	7.16	Upregulation
hsa-miR-483-3p	196	6.25	8.21	7.56	Upregulation
hsa-miR-144-3p	270635	5.47	6.96	7.71	Upregulation
hsa-miR-4732-3p	1288	3.90	6.80	8.81	Upregulation
hsa-miR-451a	669963	6.99	9.70	10.21	Upregulation

Table S3. Previously reported miRNAs involved in erythropoiesis identified in this study.

miRNA	Expression	Reference
hsa-mir-155-5p	Downregulated	[9]
hsa-mir-181a-3p	Downregulated	[20]
hsa-mir-222-3p	Downregulated	[89,90]
hsa-mir-10b-5p	Downregulated	[91]
hsa-mir-223-5p	Downregulated	[92]
hsa-mir-221-3p	Downregulated	[89,90]
hsa-mir-146a-5p	Downregulated	[9]
hsa-mir-19b-1-5p	Downregulated	[93]
hsa-mir-125a-3p	Downregulated	[94]
hsa-mir-146b-3p	Downregulated	[95]
hsa-mir-1255b-5p	Upregulated	[96]
hsa-mir-339-3p	Upregulated	[97]
hsa-mir-210-3p	Upregulated	[98]
hsa-mir-151a-5p	Upregulated	[99]
hsa-mir-22-3p	Upregulated	[99,100]
hsa-mir-16-5p	Upregulated	[93]
hsa-mir-486-3p	Upregulated	[97,31]
hsa-mir-144-5p	Upregulated	[101–103,67]
hsa-mir-96-5p	Upregulated	[32,41,104]
hsa-mir-451a	Upregulated	[22, 97,101,105]

Table S4. Molecular functions and pathways of the upregulated genes targeted by multiple downregulated miRNAs.

Genes	Molecular Functions	Functions in erythropoiesis	Number of miRNAs	miRNAs	References
AGO2	Required for RNA-mediated gene silencing (RNAi) by the RNA-induced silencing complex (RISC).	Ago2−/− mice lack fully functional red cells	24	125a-3p, 1275, 145-3p, 181b-3p, 195-3p, 221-3p, 222-3p, 223-5p, 329-3p, 3620-3p, 3690, 382-3p, 3934-5p, 3940-5p, 4420, 4510, 4665-5p, 495-3p, 605-3p, 654-5p, 671-5p, 6763-5p, 92a-1-5p, 181b-2-3p	[106]
CDKN1B	Regulator of cell cycle progression. Inhibits the kinase activity of CDK2 bound to cyclin A.	Required for red cell differentiation and enucleation	16	1185-1-3p, 148a-5p, 155-5p, 196a-5p, 196b-5p, 200c-3p, 221-3p, 221-5p, 222-3p, 323a-3p, 335-3p, 3679-5p, 455-5p, 6763-5p, 877-5p, 98-3p	[107]
MKNK2	Initiation of mRNA translation by phosphorylation of eIF4E	Not reported	15	10a-5p, 10b-5p, 134-5p, 152-5p, 181b-2-3p, 181b-3p, 1910-5p, 200c-3p, 345-3p, 3911, 4420, 4510, 4645-3p, 4802-3p, 494-3p	
SLC7A5	Import large neutral amino acids with branched or aromatic side chains	Not reported	15	10a-5p, 10b-5p, 134-5p, 152-5p, 181b-2-3p, 181b-3p, 1910-5p, 200c-3p, 345-3p, 3911, 4420, 4510, 4645-3p, 4802-3p, 494-3p	
TXNIP	Inhibits the antioxidative function of thioredoxin resulting in the accumulation of reactive oxygen species and cellular stress	Knock down affects erythroid differentiation	14	130a-5p, 134-5p, 152-5p, 212-5p, 29b-1-5p, 301b-3p, 3154, 323a-3p, 3940-5p, 4443, 4687-3p, 495-3p, 573, 7974	[108]
YOD1	Deubiquitinase activity	Not reported	14	10a-5p, 1237-3p, 1273h-3p, 196a-5p, 196b-5p, 2115-3p, 221-3p, 221-5p, 369-3p, 4455, 551b-5p, 6718-5p, 7-5p, 877-5p	
VEGFA	Growth factor active in angiogenesis, vasculogenesis, and endothelial cell growth.	Activates erythropoietin receptor	13	125a-3p, 133a-3p, 134-5p, 1-3p, 145-5p, 200c-3p,329-3p, 330-3p, 3662, 369-3p, 495-3p, 598-5p, 9-5p	
BCL2L11	Induces apoptosis and anoikis	Erythroid pro-survival factor	12	10a-5p, 10b-5p, 181b-5p, 200c-3p, 221-3p, 222-3p, 301b-3p, 4502, 4745-5p, 494-3p, 6718-5p, 9-5p	
CPEB4	CPEB4 activates mRNA translation by adding poly(A) tails to target mRNAs. Regulates activation of unfolded protein response (UPR) in the process of adaptation to ER stress. Required for cell cycle progression, specifically for cytokinesis and chromosomal segregation.	Regulates erythroid differentiation	11	145-5p, 146b-3p, 155-5p, 181b-5p, 181c-5p, 181d-5p, 382-3p, 409-3p, 487b-3p, 573, 9-5p	[109]
TUBB2A	The major constituent of microtubules.	Erythroid tubulin regulated by GATA1 and heme	11	1270, 143-3p, 181b-5p, 181c-5p, 181d-5p, 195-3p, 200c-3p, 369-3p, 432-5p, 4443, 4510	[110]

DDIT4	Regulates cell growth, proliferation, and survival via inhibition of mTORC1 by a pathway that involves DDIT4/REDD1, AKT1, the TSC1–TSC2 complex, and the GTPase RHEB. An important role in responses to cellular energy levels and cellular stress, including responses to hypoxia and DNA damage. Regulates p53/TP53-mediated apoptosis in response to DNA damage via its effect on mTORC1 activity.	Not reported	10	181b-5p, 181c-5p, 181d-5p, 200c-3p, 2115-3p, 221-3p, 222-5p, 335-3p, 3934-5p, 495-3p	
MYLIP	E3 ubiquitin-protein ligase that mediates ubiquitination and subsequent proteasomal degradation of myosin regulatory light chain (MRLC), LDLR, VLDLR, and LRP8. Activity depends on E2 enzymes of the UBE2D family.	Regulates erythroid differentiation	10	221-3p, 222-3p, 301b-3p, 323a-3p, 369-3p, 382-3p, 455-5p, 4645-3p, 654-3p, 889-3p	[111]
SLC1A5	Sodium-dependent amino acids transporter	Required for erythroid lineage determination	10	1226-3p, 125a-3p, 1273h-5p, 155-5p, 330-3p, 3934-5p, 3940-5p, 433-3p, 455-5p, 500b-3p	[112]
ZBTB7A	Transcription factor that represses the transcription of a wide range of genes involved in cell proliferation and differentiation. Involved in the switch between fetal and adult globin expression, differentiation of lymphoid progenitors into B and T lineages.	Anti-apoptotic role during terminal erythroid differentiation.	10	100-5p, 1185-1-3p, 222-3p, 296-3p, 301b-3p, 335-3p, 4443, 500b-3p, 654-5p, 877-3p	[113]

Table S5. Molecular functions and pathways of the downregulated genes targeted by multiple upregulated miRNAs.

Genes	Molecular Functions	Functions in erythropoiesis	Number of miRNAs	miRNAs	References
CCND2	Regulates cell-cycle during G (1)/S transition.	Controls erythrocyte cell number	22	106b-5p,124-3p,1248,1255b-5p,15a-5p,15b-5p,16-5p,182-5p,185-5p,29b-3p,29c-3p,301a-3p,3163,33a-3p,3613-3p,4503,4516,454-3p,610,6505-5p,6859-5p,96-5p	[114]
CDK6	Regulates cell-cycle during G (1)/S transition.	Contributes to cytoskeletal stability in erythroid cells	19	124-3p,140-3p,15a-5p,15b-5p,16-5p,183-3p,185-5p,193b-3p,218-5p,22-3p,29b-3p,29c-3p,34a-5p,362-3p,501-3p,502-3p,5091,616-5p,624-5p	[115]
RAB32	Involved in synchronization of mitochondrial fission and phagocytosis.	Affects cell proliferation and apoptosis of erythroid cells	17	124-3p,185-5p,193b-3p,3135b,339-5p,374a-5p,4646-3p,548ab,548ad-5p,548am-5p,548an,548au-5p,548ay-5p,548b-5p,548c-5p,548d-5p,6859-5p	

ZNF154	May be involved in transcriptional regulation	Not reported	14	301a-3p,3135b,454-3p,5189-5p,548ab,548ad-5p,548am-5p,548au-5p,548ay-5p,548b-5p,548c-5p,548d-5p,550a-3-5p,5690	
KLF7	Transcription factor with several functions in different cell types	Not reported	14	3163,324-5p,4301,4516,501-5p,548ab,548ad-5p,548am-5p, 548au-5p,548ay-5p,548b-5p,548c-5p, 548d-5p,6505-3p	
UBE2D1	E2 ubiquitin-conjugating enzyme involved in protein degradation through proteosome pathway	Not reported	13	192-5p,215-5p,33a-3p,513a-5p,548ab,548ac,548ad-5p,548am-5p,548au-5p,548ay-5p,548b-5p,548c-5p,548d-5p	
RBMS2	Involved in DNA replication, gene transcription, cell cycle progression, and apoptosis.	Not reported	13	194-5p,548ab,548ad-5p,548am-5p,548an,548au-5p,548ay-5p,548b-5p,548c-5p,548d-5p,550a-3-5p,5690, 6741-5p	
HOXA10	Transcription factor which is part of a developmental regulatory system	Blocks erythroid differentiation	12	144-3p,15a-5p,15b-5p,16-2-3p,16-5p,192-5p,215-5p,3611,3688-3p, 3942-5p,4791,513a-5p	[116]
ZNF711	Transcription regulator required for brain development.	Not reported	12	151a-3p,218-5p,301a-3p,454-3p,548ab,548ad-5p,548am-5p,548au-5p,548ay-5p,548b-5p,548c-5p,548d-5p	
BCL2	Blocks the apoptotic death	Overexpression delays the apoptosis of erythroid progenitors	12	15a-5p,15b-5p,16-5p,182-5p,192-5p,215-5p,29b-3p,29c-3p,30b-5p,34a-5p,451a,96-5p	[117]
USP53	Tight junction-associated protein that is involved in the survival of auditory hair cells and hearing. Maybe by modulating the barrier properties and mechanical stability of tight junctions	Not reported	12	15a-5p,15b-5p,16-5p,30b-5p,548ab,548ad-5p,548am-5p,548au-5p,548ay-5p,548b-5p,548c-5p,548d-5p	
SOX4	Transcription factor with several functions in different cell types	Not reported	11	106b-5p,151a-5p,185-3p,301a-3p,30b-5p,3163,3613-3p,4516,454-3p,548ac,6749-3p	
SULT1B1	Catalyses the sulphate conjugation of many hormones, neurotransmitters, drugs, and xenobiotic compounds.	Not reported	11	124-3p,16-2-3p,3135b,3163,3190-3p,339-5p,3611,4516,4646-3p, 616-5p,6859-5p	
GPR27	intracellular signalling cascade mediated by heterotrimeric GTP-binding proteins, or G proteins	Not reported	11	15a-5p,15b-5p,16-5p,548ab,548ad-5p,548am-5p,548au-5p,548ay-5p,548b-5p,548c-5p,548d-5p	
CMTM6	Regulates T cell activation and antitumor responses.	Not reported	10	101-3p,144-3p,16-2-3p,192-5p,215-5p,3136-5p,532-3p,548ac,660-3p,6749-3p,	
PRRG4	May control axon guidance across the CNS. Prevents the delivery of ROBO1 at the cell surface and downregulates its expression	Not reported	10	106b-5p,1248,192-5p,301a-3p,362-5p,454-3p,4659b-3p,483-3p,500a-5p,660-3p	
WNK3	Serine/threonine kinase that regulates electrolyte homeostasis, cell signaling, survival and proliferation. Acts as an	Not reported	10	106b-5p,151a-3p,15a-5p,15b-5p,16-5p,301a-3p,3613-3p,362-3p,454-3p,660-5p	

	activator and inhibitor of sodium-coupled chloride cotransporters and potassium-coupled chloride cotransporters respectively			
CBX2	Component of a Polycomb group (PcG) multiprotein PRC1-like complex, a complex class required to maintain the transcriptionally repressive state of many genes	Not reported	10	124-3p,15a-5p,15b-5p,16-5p,29b-3p,29c-3p,30b-5p,362-3p,584-3p,7976
MAP3K9	Serine/threonine kinase acts as an essential component of the MAP kinase signal transduction pathway. Plays an important role in the cascades of cellular responses evoked by changes in the environment	Not reported	10	1248,190a-5p,218-5p,301a-3p,3158-3p,339-5p,34a-5p,454-3p,500a-5p,6734-5p
SETD7	Histone methyltransferase t for epigenetic transcriptional activation.	Not reported	10	151a-3p,4659b-3p,548ab,548ad-5p,548am-5p,548au-5p,548ay-5p,548b-5p,548c-5p,548d-5p
ITGB8	Extracellular matrix protein binding	Not reported	10	3613-3p,548ab,548ad-5p,548am-5p, 548au-5p,548ay-5p,548b-5p,548c-5p, 548d-5p,590-5p

Table S6. Real-time PCR analysis after miRNA KO in HUDEP2 cells. ΔCt= Ct of target-Ct of U6 (control). ΔΔCt= ΔCt in the KO cells-ΔCt in the control cells. FC= fold change in the expression calculated by the $2^{-\Delta\Delta CT}$ method. * When the Ct values were undermined due to very low expression, a value of 40 was denoted for calculation.

d-sgRNA	miRNA	Target Ct		U6 Ct		ΔCt		ΔΔCt		FC	
		Ct 1	Ct 2	Ct 1	Ct 2	Δ Ct1	Δ Ct 2	ΔΔ Ct1	ΔΔ Ct2	FC1	FC2
d-sgRNA1	miR-182	40.0*	40.0*	10.1	10.0	29.9	30.0	13.45	14.433	0.000089	0.0000452
d-sgRNA2	miR-182	29.9	30.1	8.0	7.8	21.9	22.2	5.524	6.71	0.0217325	0.00955189
Scrambled	miR-182	25.5	25.5	9.1	10.0	16.4	15.5	0	0	1	1
d-sgRNA1	miR-144	29.4	29.7	9.3	9.3	20.0	20.4	13.28	11.77	0.0001	0.00028
d-sgRNA2	miR-144	35.6	36.1	9.3	9.4	26.3	26.7	19.64	18.03	0.0000012	0.0000037
Scrambled	miR-144	17.3	17.1	9.1	10.0	8.2	7.1	0	0	1	1
d-sgRNA1	miR-183	35.1	35.0	9.3	9.4	25.9	25.7	9.452	8.766	0.0014	0.0022
d-sgRNA2	miR-183	39.5	38.4	9.5	9.5	30.0	28.9	12.71	12.89	0.00014	0.000131
Scrambled	miR-183	26.2	26.2	9.1	10.0	17.1	16.2	0	0	1	1
d-sgRNA1	miR-451	40.0*	40.0*	9.6	9.7	30.4	30.3	22.402	22.91	0.00000018	1.2688E-07
Scrambled	miR-451	17.1	17.4	9.1	10.0	8.0	7.4	0	0	1	1
d-sgRNA1	miR-96	39.1	40.0*	5.2	5.0	33.9	34.1	17.573	18.315	0.000005128	3.06562E-06
Scrambled	miR-96	25.4	25.8	9.1	10.0	16.3	15.8	0	0	1	1
d-sgRNA1	miR-4732	32.4	32.8	9.6	9.7	22.9	23.2	11.686	11.11	0.000303503	0.000452122
Scrambled	miR-4732	22.1	22.3	10.9	10.3	11.2	12.0	0	0	1	1
d-sgRNA1	miR-215	38.7	38.7	10.4	10.4	28.4	28.3	8.46	7.839	0.002839788	0.004367427
Scrambled	miR-215	30.8	30.7	10.9	10.3	19.9	20.5	0	0	1	1

d-sgRNA1	miR-486	27.0	27.0	9.0	9.1	18.0	17.9	10.514	9.439	0.000683865	0.001440714
Scrambled	miR-486	18.5	18.8	10.9	10.3	7.5	8.5	0	0	1	1
d-sgRNA1	miR-496	35.5	34.9	10.3	9.3	25.2	25.6	7.898	8.032	0.004192418	0.003820564
Scrambled	miR-496	28.3	27.9	10.9	10.3	17.3	17.6	0	0	1	1
d-sgRNA1	miR-430	25.5	25.4	9.5	10.4	16.0	15.0	6.639	5.245	0.010033711	0.026369228
Scrambled	miR-430	20.3	20.0	10.9	10.3	9.3	9.7	0	0	1	1
